# On the Entropy of Events under Eventually Global Inflated or Deflated Probability Constraints. Application to the Supervision of Epidemic Models under Vaccination Controls

**DOI:** 10.3390/e22030284

**Published:** 2020-02-29

**Authors:** Manuel De la Sen, Asier Ibeas, Raul Nistal

**Affiliations:** 1 Institute of Research and Development of Processes IIDP, University of the Basque Country, Campus of Leioa, 48940 Leioa, Spain; raul.nistal@gmail.com; 2 Department of Telecommunications and Systems Engineering, Universitat Autònoma de Barcelona (UAB), 08193 Barcelona, Spain; Asier.Ibeas@uab.cat

**Keywords:** Shannon entropy, complete/incomplete systems of events, probabilistic uncertainty, probabilistic inflated/deflated probability constraints, epidemic model, vaccination controls, treatment controls

## Abstract

This paper extends the formulation of the Shannon entropy under probabilistic uncertainties which are basically established in terms or relative errors related to the theoretical nominal set of events. Those uncertainties can eventually translate into globally inflated or deflated probabilistic constraints. In the first case, the global probability of all the events exceeds unity while in the second one lies below unity. A simple interpretation is that the whole set of events losses completeness and that some events of negative probability might be incorporated to keep the completeness of an extended set of events. The proposed formalism is flexible enough to evaluate the need to introduce compensatory probability events or not depending on each particular application. In particular, such a design flexibility is emphasized through an application which is given related to epidemic models under vaccination and treatment controls. Switching rules are proposed to choose through time the active model, among a predefined set of models organized in a parallel structure, which better describes the registered epidemic evolution data. The supervisory monitoring is performed in the sense that the tested accumulated entropy of the absolute error of the model versus the observed data is minimized at each supervision time-interval occurring in-between each two consecutive switching time instants. The active model generates the (vaccination/treatment) controls to be injected to the monitored population. In this application, it is not proposed to introduce a compensatory event to complete the global probability to unity but instead, the estimated probabilities are re-adjusted to design the control gains.

## 1. Introduction

Classical entropy is a state function in Thermodynamics. The originator of the concept of entropy was the celebrated Rudolf Clausius in the mid-nineteenth century. The property that reversible processes have a zero variation of entropy among equilibrium states while irreversible processes have an increase of entropy is well-known. Reversible processes only occur under ideal theoretical modelling of isolated processes without energy losses such as, for instance, the Carnot cycle. Real processes are irreversible because the above ideal conditions are impossible to fulfil. In classical Thermodynamics, the Clausius equality establishes that the entropy variation in reversible cycle processes is zero. That is, the entropy variation is path-independent since it is a state function which takes an identical value at a final state being coincident with an initial one state. However, in irreversible ones the entropy variation is positive. This is the motivating reason which associates a positive variation of entropy to a “disorder increase”. It has to be pointed out that, in order to interpret correctly the non-negative variations of entropy, the system under consideration has to be an isolated one. In other words, some of the mutually interacting subsystems of an isolated system can exhibit negative variations of entropy. Later on Boltzmann, Gibbs and Maxwell have defined entropy under a statistical framework. On the other hand, Shannon entropy (named after Claude E. Shannon, 1916–2001, “the father of information theory”) is a very important tool to measure the amount of uncertainty in processes characterized in the probabilistic framework by sets of events, [1,2,3,4,5]. In some extended studies in the frameworks of physics, economy or fractional calculus, it is admitted the existence of events with negative probabilities, [4,5,6]. Entropy may be interpreted as information loss [1,2,3,7,8,9] and is useful, in particular, to characterize dynamic systems from this point of view [8]. On the other hand, entropy tools have also being used either to model or for complementary modelling support to evaluate certain epidemic models, [10,11,12,13,14,15,16,17]. In some of the studies, the investigation of epidemic models, which are ruled by a differential system of coupled equations involving the various subpopulations, has been proposed in the framework of a patchy environment [13]. The model uncertainty amount is evaluated in such a way that the time-derivative of the entropy is shown to be non-negative at a set of testing sampling instants. In [9], a technique to develop a formal Shannon entropy in the complex framework is proposed. In this way, the components of the overall entropy are calculated so as to determine the real and the imaginary parts of the state complex Shannon entropy as a natural quantum-amplitude generalization of the classical Shannon entropy.

Epidemic evolution has been typically studied through models based either on differential equations, difference equations or mixed hybrid models. Such models, because of their structure, become very appropriate to study the equilibrium points, the oscillatory behaviors, the illness permanence and the vaccination and treatment controls. See, for instance, [18,19,20,21,22,23,24] and some of the references therein. More recently, entropy-based models have been proposed for epidemic models. See, for instance, [14,15,16,25] and some of the references therein. In particular, entropy tools for analysis are mixed with differential-type models in [16,25,26]. Also, the control techniques are appropriate for studies of alternative biological problems, [27,28,29] and, in particular, for implementation of decentralized control techniques in patchy environments where several nodes are interlaced, [27,29]. Under that generic framework, it can be described, for instance, the situation of several towns with different own health centers, where the controls are implemented, and whose susceptible and infectious populations interact through in-coming and out-coming population fluxes. This paper proposes a supervisory design tool to decrease the uncertainty between the observed data related to infectious disease, or those given by a complex model related to a disease, via the use of a set of dynamic integrated simplest models together with a higher-level supervisory switching algorithm. Such a hierarchical structure selects on line the most appropriate model as the one which has a smaller error uncertainty according to an entropy description (the so-called “active model”). Such an active model is re-updated through time in the sense that another active model can enter into operation. In that way, the active model is used to generate the correcting sanitary actions to control the epidemics such as, for instance, the gains of the vaccination and antiviral or antibiotic treatment controls and the corresponding control interventions.

The paper is organized as follows: Section 2 recalls some basic concepts of Shannon entropy. Also, those concepts are extended to the case of eventual presence of relative probabilistic uncertainties in the defined set of events compared to the nominal set of probabilities. Section 3 states and proves mathematical results for the Shannon entropy for the case when a nominal complete finite and discrete set of events is eventually subject to relative errors of their associate probabilities in some of all of their various integrating events. The error-free nominal system of events is assumed to be complete. The current system of events under probabilistic errors may be either deflated or inflated in the sense that the total probability for the whole sets might be either below or beyond unity, respectively, so that it might be non-complete. In the case of globally inflated of deflated probability of the whole set of events, new compensatory events can be used to accomplish with a unity global probability. The entropy of the current system is compared via quantified worst-case results to that of the nominal system. Section 4 applies partially the results of the former sections to epidemics control in the case that either the disease transmission coefficient rate is not well-known or it varies through time due, for instance, to seasonality. The controls are typically of vaccination or treatment type or appropriate mixed combinations of both of them. A finite predefined set of running models described by a system of coupled differential equations is set. Such a whole discrete set covers a range of variation of such a coefficient transmission rate within known lower-bound and upper-bound limits. Each model is driven by a constant disease transmission rate and the whole set of models covers the whole range of foreseen variation of such a parameter in the real system. Since the set of models is finite, the various values of the disease transmission rate are integrated in a discrete set within the whole range of admissible variation of the true coefficient rate. A supervisory technique of control monitoring is proposed which chooses the so-called active model which minimizes the accumulated entropy of the absolute error data/model within each supervision interval. A switching rule allows to choose another active model as soon as it is detected that the current active model becomes more uncertain than other(s) related to the observed data. The active model supplies the (vaccination and/or treatment) controls to be injected to the real epidemic process. Due to the particular nature of the problem, compensatory events are not introduced for equalizing the global probability to unity. Instead, a re-adjustment of the error probabilities related to the true available data is performed to calculate the control gains provided by the active model. It can be pointed out that some parameters of the epidemic description evolutions, typically the coefficient transmission rate, can vary according to seasonality [30]. This justifies the use of simpler active invariant models to describe the epidemics evolution along time subject to appropriate model switching. On the other hand, the existing medical tests which evaluate the proportions of healthy and infected individuals within the total population are not always subject to confluent worst-case estimation errors. See, for instance [31,32]. In the case that the estimated global probability of the various subpopulations is not unity, the estimated worst-case probabilities need to be appropriately amended before an intervention. This design work is performed and discussed in Section 5 through numerical simulations in confluence with the above mentioned supervision monitoring technique. Finally, conclusions end the paper.

## 2. Basic Entropy Preliminaries

Assume a finite system of events $A=\left\{A_{i}:i\in \bar{n}\right\}$ of respective probabilities $p_{i}$ for $A_{i}$; $i\in \bar{n}=\left\{1,2,\cdots ,n\right\}$ with $\left\{p_{i}:i\in \bar{n}\right\}\subset \left[0,\;1\right]$, with $\sum_{i=1}^{n}p_{i}=1$ which is complete, that is, only one event occurs at each trial (e.g., the appearance of 1 to 6 points in rolling a die). The Shannon entropy is defined as follows:

(1) $$H\left(p_{1},p_{2},\ldots ..p_{n}\right)=-\sum_{i=1}^{n}p_{i}\ln p_{i}$$

which serves as a suitable measure of the uncertainty of the above finite scheme. The name “entropy” pursues a physical analogy with parallel problems, for instance, in Thermodynamics or Statistics Physics which does not have a similar sense here so that there is no need to go into in the current context. The above entropy has been defined with neperian logarithms but any logarithm with a fixed base could be used instead with no loss in generality.

Note that $H\left(p_{1},\;p_{2},\ldots ..p_{n}\right)\geq 0$ with $H\left(p_{1},\;p_{2},\ldots ..p_{n}\right)=0$ if and only if $p_{j}=1$ for some arbitrary $j\in \bar{n}$ and $p_{i}=0$; $\forall i\left(\neq j\right)\in \bar{n}$ and

$$H_{\max}=H\left(p,\;p,\ldots .,p\right)=H\left(1/n,\;1/n,\ldots .,1/n\right)=\max_{p_{i}\in \left[0,\;1\right],\;\sum_{i=1}^{n}p_{i}=1}=-nplnp=-ln\left(1/n\right)=lnn\geq 0$$

$$H_{\max}=H\left(p,\;p,\ldots .,p\right)=H\left(1/n,\;1/n,\ldots .,1/n\right)=\max_{p_{i}\in \left[0,\;1\right],\;\sum_{i=1}^{n}p_{i}=1}=-nplnp=-ln\left(1/n\right)=lnn\geq 0$$

with $H_{\max}=0$ if and only if n=p=1. Assume that the probabilities are uncertain, given by $p_{i}\left(1+\varepsilon_{i}\right)$; $\forall i\in \bar{n}$, subject to the constraints $p_{i}\left(1-{\bar{\varepsilon }}_{i0}\right)\leq p_{i}\left(1+\varepsilon_{i}\right)\leq p_{i}\left(1+{\bar{\varepsilon }}_{i1}\right)$; $\forall i\in \bar{n}$, where ${\bar{\varepsilon }}_{i0}$ and ${\bar{\varepsilon }}_{i1}$ are known so that the current entropy (or the entropy of the current system) is uncertain and given by $H_{\varepsilon }=H\left(p_{1}\left(1+\varepsilon_{1}\right),p_{2}\left(1+\varepsilon_{2}\right),\ldots ..p_{n}\left(1+\varepsilon_{n}\right)\right)$; $\forall i\in \bar{n}$. The reference entropy $H_{*}=H\left(p_{1},p_{2},\ldots ..p_{n}\right)$ for the case when the probabilities of all the events are precisely known being equal to $p_{i}$; $\forall i\in \bar{n}$ is said to be the nominal entropy (or the entropy of the nominal system). Some constraints have to be fulfilled in order for the formulation to be coherent related to the entropy bounds under probabilistic constraints in the set of involved events. The following related result follows:

**Lemma** **1.** *Assume a nonempty finite complete nominal system of events* $A_{*}=\left\{A_{i*}:i\in \bar{n}\right\}$ *of respective nominal probabilities* $p_{i}\in \left[0,\;1\right]$ *for* $A_{i*}$ *;* $\forall i\in \bar{n}=\left\{1,2,\cdots ,n\right\}$ *and a current (or uncertain) version of the system of events* $A=\left\{A_{i}:i\in \bar{n}\right\}$ *of uncertain probabilities* $p_{i}\left(1+\varepsilon_{i}\right)\in \left[0,1\right]$ *;* $\forall i\in \bar{n}$ *, where* $\varepsilon_{i}\in R$ *are relative probability errors due to probabilistic uncertainties. Define the following disjoint subsets of* A *:*

$A_{+}=\left\{A_{i}\in A:\;\varepsilon_{i}>0\right\}$ *,* $A_{-}=\left\{A_{i}\in A:\;\varepsilon_{i}<0\right\}$ *,* $A_{0}=\left\{A_{i}\in A:\;\varepsilon_{i}=0\right\}$ *.*

*Then, the following constraints hold:*

***(i)***  A *is complete if and only if* $\sum_{i=1}^{n}\varepsilon_{i}p_{i}=0$ *(global probabilistic uncertainty mutual compensation)*
***(ii)***  $max\;\left(cardA_{+},\;cardA_{-}\right)<n$ *, and* $min\;\left(cardA_{+},\;cardA_{-}\right)>0$ *or* $cardA_{0}=n$ *(and then* $A_{+}=A_{-}=\emptyset$ *)*

**Proof:**  The proof of Property (i) follows since $A_{*}$ being complete implies that A is complete if and only if $\sum_{i=1}^{n}p_{i}\left(1+\varepsilon_{i}\right)=1$.

$\sum_{i=1}^{n}p_{i}\left(1+\varepsilon_{i}\right)=1=\sum_{i=1}^{n}p_{i}+\sum_{i=1}^{n}p_{i}\varepsilon_{i}=1+\sum_{i=1}^{n}p_{i}\varepsilon_{i}\Leftrightarrow \sum_{i=1}^{n}p_{i}\varepsilon_{i}=0$

and A is complete since $\sum_{i=1}^{n}p_{i}\left(1+\varepsilon_{i}\right)=1$. Property (i) has been proved, The proof of Property (ii) follows from Property (i). To prove the first constraint, assume, on the contrary, that $max\;\left(cardA_{+},\;cardA_{-}\right)=n$. Then, either $cardA_{+}=n$ and $A_{-}=A_{0}=\emptyset$ or $cardA_{-}=n$ and $A_{+}=A_{0}=\emptyset$. Assume that $cardA_{+}=n$ and $A_{-}=A_{0}=\emptyset$. Then, $\sum_{i=1}^{n}\varepsilon_{i}p_{i}>0$ which contradicts Property (i). Similarly, if $cardA_{-}=n$ and $A_{+}=A_{0}=\emptyset$ then $\sum_{i=1}^{n}\varepsilon_{i}p_{i}<0$ which again contradicts Property (i). Thus, $max\;\left(cardA_{+},\;cardA_{-}\right)<n$ and the first constraint of Property (i) has been proved. Now, assume that $min\;\left(cardA_{+},\;cardA_{-}\right)=0$ and $0\leq cardA_{0}<n$. First, assume that $cardA_{-}=0$ and $cardA_{+}=cardA-cardA_{0}=max\;\left(cardA_{+},\;cardA_{-}\right)\leq n$ (from the already proved above first constraint of this property). If $cardA_{+}>0$ then, $\sum_{i=1}^{n}\varepsilon_{i}p_{i}>0$ which contradicts Property (i). If $cardA_{+}=0$ then $A=A_{0}$ and $cardA_{0}=n$. So, if $cardA_{-}=0$ then $cardA_{+}=0$ and $cardA_{0}=n$ (then $A=A_{0}$). In the same way, interchanging the roles of $A_{+}$ and $A_{-}$, it follows that, if $cardA_{+}=0$ then $cardA_{-}=0$ and $cardA_{0}=n$. One concludes that either $min\;\left(cardA_{+},\;cardA_{-}\right)>0$ or $cardA_{0}=n$ and Property (ii) is proved.

Note that, in Lemma 1, $A_{0}\subset A$ is the subset of probabilistically certain events of A (in the sent that is elements have a known probability) and $A_{+}\cup A_{-}\subset A$ is the subset of probabilistically uncertain events of A. For A being nonempty, any of the sets $A_{0}$, $A_{+}$ and $A_{-}$ or pair combinations may be empty. Note also that the probabilistic uncertainties have been considered with fixed values $\varepsilon_{i}\in \left[0,1\right]$; $\forall i\in \bar{n}$. □

## 3. Entropy Versus Global Inflated and Deflated Probability Constraints in Incomplete Systems of Events under Probabilistic Uncertainties

An important issue to be addressed is how to deal with the case when, due to incomplete knowledge of the probabilistic uncertainties, the sum of probabilities of the current complete system of events, i.e., that related to the uncertain values of individual probability values exceeds unity, that is $\sum_{i=1}^{n}p_{i}\left(1+\varepsilon_{i}\right)>1$, a constraint referred to as “global inflated probability”. If both the nominal individual and nominal global probability constraints, $p_{i}\geq 0$; $\forall i\in \bar{n}$ and $\sum_{i=1}^{n}p_{i}=1$ hold then there is a global inflated probability if and only if the probabilistic disturbances fulfill $\sum_{i=1}^{n}p_{i}\varepsilon_{i}>0$. It is always possible to include the case of “global deflated probability” if $\sum_{i=1}^{n}p_{i}\left(1+\varepsilon_{i}\right)<1$ implying that $\sum_{i=1}^{n}p_{i}\varepsilon_{i}<0$ provided that $\sum_{i=1}^{n}p_{i}=1$.

Three possible solutions to cope with this drawback, associate to an exceeding amount of modeled probabilistic uncertainty leading to global inflated probability, are:

(a) To incorporate a new (non empty) event $A_{n+1}$ with negative probability which reduces the probabilistic uncertainty so that the global probability constraint of the extended complete system of events $A_{e}=A\cup A_{n+1}$ holds. Obviously, A losses is characteristic property of being a complete system of events and $A_{e}$ is said to be partially complete since it fulfills the global probability constraint but it has one negative probability.

(b) To modify all or some of the probabilistic uncertainty relative amounts $\varepsilon_{i}$; $i\in \bar{n}$ so that the global probability constraint of the complete system of events holds for the new amended fixed set $\left\{\varepsilon_{i}:\;i\in \bar{n}\right\}$.

(c) To consider uncertain normalized probabilities $p_{i}^{´}=\frac{p_{i}\left(1+\varepsilon_{i}\right)}{\sum_{i=1}^{n}p_{i}\left(1+\varepsilon_{i}\right)}$; $i\in \bar{n}$. In this case, there is no need to modify the individual uncertainties of the events but the initial uncertainties are kept. Furthermore, the events would keep its ordination according to uncertainties, namely, if $p_{i}\left(1+\varepsilon_{i}\right)\leq p_{j}\left(1+\varepsilon_{j}\right)$ for $i,j\left(\neq i\right)\in \bar{n}$ then $p_{i}^{´}\leq p_{j}^{´}$, the modified set of events is complete and keeps the same number of events as the initial one.

Two parallel solutions to cope with global deflated probability are:

(d) To incorporate a new (non empty) event $A_{n+1}$ with positive probability which decreases the probabilistic uncertainty so that the global probability constraint of the extended complete system of events $A_{e}=A\cup A_{n+1}$ holds. As before, the current system of events A losses is completeness and $A_{e}$ is complete. 

(e) To modify all or some of the probabilistic uncertainty relative amounts $\varepsilon_{i}$; $i\in \bar{n}$ so that new amended fixed set $\left\{\varepsilon_{i}:\;i\in \bar{n}\right\}$ agrees with the global probability constraint.

Firstly, we note that the introduction of negative probabilities invoked in the first proposed solution has a sense in certain problems. In this context, Dirac commented in a speech in 1942 that negative probabilities can have a sense in certain problems as negative money has in some financial situations. Later on, Feynman has used also this concept to describe some problems of physics [4] and also said that negative trees have nonsense but negative probability can have sense as negative money has in some financial situations. More recently, Tenreiro-Machado has used this concept in the context of fractional calculus [6].

It is now proved in the next result that the incorporation of a new event of negative probability to define from the system of events A an extended partial complete system of events $A_{e}$ does not alterate the property of the non-negativity of the Shannon entropy in the sense that that of the extended system of events is still non-negative. At the same time, it is proved that 

(1) If $1<\sum_{i=1}^{n}p_{i}\left(1+\varepsilon_{i}\right)\leq 2$ then the real part of the entropy of $A_{e}$ (which is complex because of the negative probability of the added event for completeness) is smaller that that of A. The interpretation is that the excessive disorder in A, due to its global inflated probability, is reduced (equivalently, “the order amount” $A_{e}$ is increased in with respect to A) when building its extended version $A_{e}$ by the contribution of the added event of negative probability. Also, both A and $A_{e}$ are not complete while $A_{e}$ is partially complete since it fulfills the global probability constraint although not the partial ones in all the events due to the incorporated one of negative probability.

(2) If $\sum_{i=1}^{n}p_{i}\left(1+\varepsilon_{i}\right)>2$ then the above qualitative considerations on increase/ decrease of “order” or” disorder” are reversed with respect to Case a.

**Theorem** **1.** *Assume a nonempty finite complete nominal system of events*$A_{*}=\left\{A_{i*}:i\in \bar{n}\right\}$*of respective nominal probabilities*$p_{i}\in \left[0,\;1\right]$*for*$A_{i*}$*;*$\forall i\in \bar{n}=\left\{1,2,\cdots ,n\right\}$*and a current (or uncertain) version of the system of events*$A=\left\{A_{i}:i\in \bar{n}\right\}$*of uncertain probabilities*$p_{i}\left(1+\varepsilon_{i}\right)\in \left[0,1\right]$*;*$\forall i\in \bar{n}$*. Assume that*$2\geq \sum_{i=1}^{n}p_{i}\left(1+\varepsilon_{i}\right)>1$*(that is, there is a global inflated probability of the current system of events). Define the extended system of events*$A_{e}=A\cup A_{n+1}$*such that*$A_{n+1}$*has a probability*$p_{n+1}=p_{n+1}\left(\varepsilon_{1},\;\varepsilon_{2},\cdots ,\varepsilon_{n}\right)=1-\sum_{i=1}^{n}p_{i}\left(1+\varepsilon_{i}\right)$*. Then,*

***(i)***  *If*$2\geq \sum_{i=1}^{n}p_{i}\left(1+\varepsilon_{i}\right)>1$*then*$ReH_{\varepsilon 1}\leq H_{\varepsilon }$*with*$0\leq ReH_{\varepsilon 1}=H_{\varepsilon }$*if and only if*$\sum_{i=1}^{n}p_{i}\left(1+\varepsilon_{i}\right)=2$*.*
***(ii)***  *If*$\sum_{i=1}^{n}p_{i}\left(1+\varepsilon_{i}\right)>2$*then*$H_{\varepsilon 1}>H_{\varepsilon }\geq 0$*,*

where:

(2) $$H_{\varepsilon }=H\left(p_{1}\left(1+\varepsilon_{1}\right),p_{2}\left(1+\varepsilon_{2}\right),\ldots ..,p_{n}\left(1+\varepsilon_{n}\right)\right)=\sum_{i=1}^{n}p_{i}\left(1+\varepsilon_{i}\right)\left(lnp_{i}+ln\left(1+\varepsilon_{i}\right)\right)$$

(3) $$\begin{array}{c} H_{\varepsilon 1}=H\left(p_{1}\left(1+\varepsilon_{1}\right),p_{2}\left(1+\varepsilon_{2}\right),\ldots ..,p_{n}\left(1+\varepsilon_{n}\right),\;p_{n}\right)\\ =H\left(p_{1}\left(1+\varepsilon_{1}\right),p_{2}\left(1+\varepsilon_{2}\right),\ldots ..,p_{n}\left(1+\varepsilon_{n}\right),\;1-\sum_{i=1}^{n}p_{i}\left(1+\varepsilon_{i}\right)\right) \end{array}$$

*are the respective entropies of* A *and* $A_{e}$*, the second one being complex.*

**Proof:**  Since $\sum_{i=1}^{n}p_{i}\left(1+\varepsilon_{i}\right)>1$ then $A=\left\{A_{i1}:i\in \bar{n}\right\}$ is not complete. Since the nominal system of events is complete then $\sum_{i=1}^{n}p_{i}=1$ with $p_{i}\geq 0$; $\forall i\in \bar{n}$ and $p_{n+1}=1-\sum_{i=1}^{n}p_{i}\left(1+\varepsilon_{i}\right)<0$. The entropy of A is

and that of the extended system of events $A_{e}$ is $H_{\varepsilon 1}$. Consider two cases:

*Case* (*a*) $0<\sum_{i=1}^{n}p_{i}\left(1+\varepsilon_{i}\right)-1\leq 1$

Since the neperian logarithm of a negative real number exists in the complex field and it is real such that its real part is the neperian logarithm of its modulus and the imaginary part is iπ ($i=\sqrt{-1}$ being the imaginary unit), so that it becomes a negative real number, one gets the following set of relations:

(4) $$\begin{array}{c} -H_{\varepsilon 1}=\sum_{i=1}^{n}p_{i}\left(1+\varepsilon_{i}\right)ln\left(p_{i}\left(1+\varepsilon_{i}\right)\right)+\left(1-\sum_{i=1}^{n}p_{i}\left(1+\varepsilon_{i}\right)\right)\;ln\left(1-\sum_{i=1}^{n}p_{i}\left(1+\varepsilon_{i}\right)\right)\\ =-H_{\varepsilon }+\left(1-\sum_{i=1}^{n}p_{i}\left(1+\varepsilon_{i}\right)\right)\;ln\left(1-\sum_{i=1}^{n}p_{i}\left(1+\varepsilon_{i}\right)\right)\\ =-H_{\varepsilon }-\left(\sum_{i=1}^{n}p_{i}\left(1+\varepsilon_{i}\right)-1\right)\;ln\left(1-\sum_{i=1}^{n}p_{i}\left(1+\varepsilon_{i}\right)\right)\\ =-H_{\varepsilon }-\left(\sum_{i=1}^{n}p_{i}\left(1+\varepsilon_{i}\right)-1\right)\;\left(ln\;\left|1-\sum_{i=1}^{n}p_{i}\left(1+\varepsilon_{i}\right)\right|+i\pi \right)\\ =-H_{\varepsilon }+\left|\sum_{i=1}^{n}p_{i}\left(1+\varepsilon_{i}\right)-1\right|\;\left(\left|ln\;\left|1-\sum_{i=1}^{n}p_{i}\left(1+\varepsilon_{i}\right)\right|\right|+i\pi \right) \end{array}$$

Then:

(5) $$Re\;\left(-H_{\varepsilon 1}\right)=-H_{\varepsilon }+\left|\left(\sum_{i=1}^{n}p_{i}\left(1+\varepsilon_{i}\right)-1\right)ln\;\left|\sum_{i=1}^{n}p_{i}\left(1+\varepsilon_{i}\right)-1\right|\right|$$

since $0<\sum_{i=1}^{n}p_{i}\left(1+\varepsilon_{i}\right)-1\leq 1$ leads to

(6) $$ln\;\left|\sum_{i=1}^{n}p_{i}\left(1+\varepsilon_{i}\right)-1\right|\leq 0,\;ln\left(1-\sum_{i=1}^{n}p_{i}\left(1+\varepsilon_{i}\right)\right)=-ln\left|1-\sum_{i=1}^{n}p_{i}\left(1+\varepsilon_{i}\right)\right|>0$$

One concludes that that $ReH_{\varepsilon 1}\leq H_{\varepsilon }$ with $0\leq ReH_{\varepsilon 1}=H_{\varepsilon }$ if and only if $\sum_{i=1}^{n}p_{i}\left(1+\varepsilon_{i}\right)=2$.

It has to be proved now that $ReH_{\varepsilon 1}\geq 0$. Assume, on the contrary, that $ReH_{\varepsilon 1}<0$. Since $ReH_{\varepsilon 1}<H_{\varepsilon }$, this happens if and only if the following contradiction holds

(7) $$\begin{array}{c} \sum_{i=1}^{n}p_{i}\left(1+\varepsilon_{i}\right)\left(lnp_{i}+ln\left(1+\varepsilon_{i}\right)\right)=\sum_{i=1}^{n}ln\left[{\left(p_{i}\left(1+\varepsilon_{i}\right)\right)}^{p_{i}\left(1+\varepsilon_{i}\right)}\right]=ln\;\left[\prod_{i=1}^{n}{\left(p_{i}\left(1+\varepsilon_{i}\right)\right)}^{p_{i}\left(1+\varepsilon_{i}\right)}\right]\\ =H_{\varepsilon }<\left(\sum_{i=1}^{n}p_{i}\left(1+\varepsilon_{i1}\right)-1\right)\left|ln\;\left|\sum_{i=1}^{n}p_{i}\left(1+\varepsilon_{i1}\right)-1\right|\right|=\left(\sum_{i=1}^{n}p_{i}\varepsilon_{i1}\right)ln\left|\sum_{i=1}^{n}p_{i}\varepsilon_{i1}\right|\\ =\left(\sum_{i=1}^{n}p_{i}\varepsilon_{i1}\right)\left(ln\sum_{i=1}^{n}p_{i}\varepsilon_{i1}\right)=ln\;\left[{\left(\sum_{i=1}^{n}p_{i}\varepsilon_{i1}\right)}^{\sum_{i=1}^{n}p_{i}\varepsilon_{i1}}\right] \end{array}$$

since $\sum_{i=1}^{n}p_{i}\left(1+\varepsilon_{i}\right)>1$ in the assumption of global inflated probability. Then, $0\leq ReH_{\varepsilon 1}\leq H_{\varepsilon }$.

*Case* (*b*) $1<\sum_{i=1}^{n}p_{i}\left(1+\varepsilon_{i}\right)-1\leq z$ for some $z\left(>1\right)\in R_{+}$. Then $ln\;\left|\sum_{i=1}^{n}p_{i}\left(1+\varepsilon_{i}\right)-1\right|>0$ and then the last above identity changes the second term right-hand-side term resulting in:

$-H_{\varepsilon 1}=Re\;\left(-H_{\varepsilon 1}\right)=-H_{\varepsilon }-\left|\sum_{i=1}^{n}p_{i}\left(1+\varepsilon_{i}\right)-1\right|\;\left|ln\left(\sum_{i=1}^{n}p_{i}\left(1+\varepsilon_{i}\right)-1\right)\right|$

(8) $$ReH_{\varepsilon 1}=H_{\varepsilon }+\left|\left(\sum_{i=1}^{n}p_{i}\left(1+\varepsilon_{i}\right)-1\right)ln\;\left|\sum_{i=1}^{n}p_{i}\left(1+\varepsilon_{i}\right)-1\right|\right|>H_{\varepsilon }\geq 0$$

□

It is now proved in the next result for the case of deflated probability that the introduction of an additional event of positive probability to define from the system of events A an extended partial complete system of events $A_{e}$ does not modify the property of the non-negativity of the Shannon entropy in the sense that that of the extended system of events is still non-negative. At the same time, it is proved that the entropy of $A_{e}$ is larger that that of A. The interpretation is that the excessively low disorder in A, due to its global deflated probability, is increased (equivalently, “the order amount” is decreased) when building its extended version $A_{e}$ by the contribution of the added event $A_{n+1}$ of positive probability. Also, A is not complete while $A_{e}$ is complete.

**Theorem** **2.** *Assume a nonempty finite complete nominal system of events* $A_{*}=\left\{A_{i*}:i\in \bar{n}\right\}$ *of respective nominal probabilities* $p_{i}\in \left[0,\;1\right]$ *for* $A_{i*}$ *;* $\forall i\in \bar{n}=\left\{1,2,\cdots ,n\right\}$ *and a current (or uncertain) version of the system of events* $A=\left\{A_{i}:i\in \bar{n}\right\}$ *of uncertain probabilities* $p_{i}\left(1+\varepsilon_{i}\right)\in \left[0,1\right]$ *;* $\forall i\in \bar{n}$ *. Assume that* $\sum_{i=1}^{n}p_{i}\left(1+\varepsilon_{i}\right)<1$ *(that is, there is a global deflated probability of the current system of events). Define the extended system of events* $A_{e0}=A\cup A_{n+1}$ *such that* $A_{n+1}$ *has a probability* $p_{n+1}=p_{n+1}\left(\varepsilon_{1},\;\varepsilon_{2},\cdots ,\varepsilon_{n}\right)=1-\sum_{i=1}^{n}p_{i}\left(1+\varepsilon_{i}\right)$ *. Then,* $H_{\varepsilon 0}>H_{\varepsilon }\geq 0$ *, where:*

(9) $$H_{\varepsilon }=H\left(p_{1}\left(1+\varepsilon_{1}\right),p_{2}\left(1+\varepsilon_{2}\right),\ldots ..,p_{n}\left(1+\varepsilon_{n}\right)\right)=\sum_{i=1}^{n}p_{i}\left(1+\varepsilon_{i}\right)\left(lnp_{i}+ln\left(1+\varepsilon_{i}\right)\right)$$

(10) $$\begin{array}{c} H_{\varepsilon 0}=H\left(p_{1}\left(1+\varepsilon_{1}\right),p_{2}\left(1+\varepsilon_{2}\right),\ldots ..,p_{n}\left(1+\varepsilon_{n}\right),\;p_{n+1}\right)\\ =H\left(p_{1}\left(1+\varepsilon_{1}\right),p_{2}\left(1+\varepsilon_{2}\right),\ldots ..,p_{n}\left(1+\varepsilon_{n}\right),\;\sum_{i=1}^{n}p_{i}\left(1+\varepsilon_{i}\right)-1\right) \end{array}$$

*are the respective entropies of* A *and* $A_{e0}$ *.*

**Proof:**  Since $\sum_{i=1}^{n}p_{i}\left(1+\varepsilon_{i}\right)<1$ then $A=\left\{A_{i}:i\in \bar{n}\right\}$ is not complete. Since the nominal system of events is complete then $\sum_{i=1}^{n}p_{i}=1$ with $p_{i}\geq 0$; $\forall i\in \bar{n}$ and $p_{n+1}=1-\sum_{i=1}^{n}p_{i}\left(1+\varepsilon_{i}\right)>0$. The entropy of A is $H_{\varepsilon }$ and that of $A_{e0}$ is:

(11) $$\begin{array}{c} H_{\varepsilon 0}=H\left(p_{1}\left(1+\varepsilon_{1}\right),p_{2}\left(1+\varepsilon_{2}\right),\ldots ..,p_{n}\left(1+\varepsilon_{n}\right),\;p_{n+1}\right)\\ =H\left(p_{1}\left(1+\varepsilon_{1}\right),p_{2}\left(1+\varepsilon_{2}\right),\ldots ..,p_{n}\left(1+\varepsilon_{n}\right),\;\sum_{i=1}^{n}p_{i}\left(1+\varepsilon_{i}\right)-1\right) \end{array}$$

Since $0<1-\sum_{i=1}^{n}p_{i}\left(1+\varepsilon_{i}\right)<1$, one gets the following set of relations:

(12) $$\begin{array}{c} -H_{\varepsilon 0}=\sum_{i=1}^{n}p_{i}\left(1+\varepsilon_{i}\right)ln\left(p_{i}\left(1+\varepsilon_{i}\right)\right)+\left(1-\sum_{i=1}^{n}p_{i}\left(1+\varepsilon_{i}\right)\right)\;ln\left(1-\sum_{i=1}^{n}p_{i}\left(1+\varepsilon_{i}\right)\right)\\ =-H_{\varepsilon }+\left(1-\sum_{i=1}^{n}p_{i}\left(1+\varepsilon_{i}\right)\right)\;ln\left(1-\sum_{i=1}^{n}p_{i}\left(1+\varepsilon_{i}\right)\right)\\ =-H_{\varepsilon }-\left(1-\sum_{i=1}^{n}p_{i}\left(1+\varepsilon_{i}\right)\right)\left|ln\left(1-\sum_{i=1}^{n}p_{i}\left(1+\varepsilon_{i}\right)\right)\right|<-H_{\varepsilon } \end{array}$$

One concludes that that $H_{\varepsilon 0}>H_{\varepsilon }\geq 0$.

Note that since we entropy is defined with a sum of weighted logarithms of nonnegative real numbers bounded by unity in the usual cases (which exclude negative probabilities) then the Shannon entropy of the worst case of the probability uncertainty is not necessarily larger than or equal to the nominal one. □

**Example** **1.** *Assume that the nominal system of two events is* $A=\left\{A_{1}\left(p_{1}\right),\;A_{2}\left(p_{2}\right)\right\}$ *with* $p_{1}=p_{2}=0.5$ *, then* $H_{*}=-2\times 0.5\times ln0.5=-ln0.5=0.6931$ *. Assume that it probabilistic uncertainty is given by* ε=0.1 *,* $p_{1}\left(1+\varepsilon \right)=0.5=0.55$ *,* $p_{2}\left(1-\varepsilon \right)=0.45=0.55$ *. Then, the entropy under the uncertain probability of* $A_{\varepsilon }=\left\{A_{1}\left(p_{1}\left(1+\varepsilon \right)\right),\;A_{2}\left(p_{2}\left(1-\varepsilon \right)\right)\right\}$ *is* $H_{\varepsilon }=-0.55ln\left(0.55\right)-0.45ln\left(0.45\right)=0.68814<H_{*}$ *.*

**Example** **2.** *Consider the complete system of events* $A=\left\{A_{i}:i\in \bar{n}\right\}$ *such that* $p_{i}=\lambda_{i}p$ *;* $\forall i\in \bar{n}$ *, for some* p>0 *, is the probability of* $A_{i}$ *,* $\forall i\in \bar{n}$ *and constants* $\lambda_{i}\in R_{0+}=R_{+}\cup \left\{0\right\}$ *;* $\forall i\in \bar{n}$ *satisfying that* $\sum_{i=1}^{n}\lambda_{i}=1/p$ *. Note that* $\sum_{i=1}^{n}p_{i}=1$ *so that there is no negative probability and neither global inflated or global deflated probabilities. The Shannon entropy is the everywhere continuous real concave function:*

(13) $$H_{a}=H_{a}\left(p\right)=H\left(\lambda_{1}p,\lambda_{2}p,\ldots .,\lambda_{n}p\right)=-\sum_{i=1}^{n}\lambda_{i}pln\left(\lambda_{i}p\right)$$

*whose maximum value is reached at a real constant* p* *if* ${dH/dp]}_{p=p*}=0$ *. Direct calculation yields, since* $\sum_{i=1}^{n}\lambda_{i}=1/p$ *,*

(14) $$\begin{array}{c} \frac{dH_{a}\left(p\right)}{dp}=-\sum_{i=1}^{n}\left(\lambda_{i}ln\left(\lambda_{i}p\right)+\lambda_{i}\right)=\sum_{i=1}^{n}\lambda_{i}\left(1+lnp+ln\lambda_{i}\right)\\ =-\sum_{i=1}^{n}\left(\lambda_{i}\left(1+lnp\right)+ln\left({\lambda_{i}}^{\lambda_{i}}\right)\right)=-\frac{1+lnp}{p}-\sum_{i=1}^{n}\left(ln\left({\lambda_{i}}^{\lambda_{i}}\right)\right)=0 \end{array}$$

*Such a*p*is unique for the given set$\left\{\lambda_{i}:i\in \bar{n}\right\}$*, since the function*$H_{a}\left(p\right)$*is concave in its whole definition domain, and admissible provided that*p*∈[0, 1]*. The unique solution for each given set*$\left\{\lambda_{i}:i\in \bar{n}\right\}$*is*$p^{*}=p^{*}\left(\lambda_{1},\;\lambda_{2},\ldots ,\lambda_{n}\right)$ satisfying the constraint:

(15) $$f\left(p*\right)=\frac{1+lnp*}{p*}=K_{\lambda }=-\sum_{i=1}^{n}\left(ln\left({\lambda_{i}}^{\lambda_{i}}\right)\right)<0$$

*Since*f:[0 ,1]→R*is continuous and bounded from above with*f(0)=−∞*and*f(1)=1*, and*K<0*such that a real*p*exists in(0, 1]*and it is unique, since there is a unique*p*such that$f\left(p*\right)=K_{\lambda }$*, for each given set*$\left\{\lambda_{i}:i\in \bar{n}\right\}$ and the maximum entropy becomes

(16) $$H_{max}=H_{a}\left(p*\right)=H\left(\lambda_{1}p*,\lambda_{2}p*,\ldots .,\lambda_{n}p*\right)=-\sum_{i=1}^{n}\lambda_{i}p*ln\left(\lambda_{i}p*\right)$$

*In particular, if* n=2 *with* $p_{1}=\lambda_{1}p$ *and* $p_{2}=\lambda_{2}p=1-\lambda_{1}p$ *then*

$H_{a}\left(p\right)=-\lambda_{1}pln\left(\lambda_{1}p\right)-\left(1-\lambda_{1}p\right)ln\left(1-\lambda_{1}p\right)$

*and:*

(17) $$\begin{array}{c} \frac{dH_{a}\left(p\right)}{dp}=-\lambda_{1}ln\left(\lambda_{1}p\right)-\lambda_{1}p\frac{\lambda_{1}}{\lambda_{1}p}+\lambda_{1}ln\left(1-\lambda_{1}p\right)-\left(1-\lambda_{1}p\right)\frac{-\lambda_{1}}{1-\lambda_{1}p}\\ =-\lambda_{1}\left(ln\left(\lambda_{1}p\right)-ln\left(1-\lambda_{1}p\right)\right)=0 \end{array}$$

*leading to*$\lambda_{2}p=1-\lambda_{1}p=1/2$*so that*$\lambda_{1}p=\lambda_{2}p=1/2$*and*$p*=1/2\lambda_{1}$*. Note that this is equivalent to*$ln\frac{\lambda_{1}p*}{1-\lambda_{1}p*}=0$*, that is*$\frac{\lambda_{1}p*}{1-\lambda_{1}p*}=1$*. In particular, if*$\lambda_{1}=\lambda_{2}=1$*then*p*=1/2*. Assume now that one uses any basis*b of logarithms to define the entropy so that:

(18) $$H_{ab}\left(p\right)=-\lambda_{1}p{log}_{b}\left(\lambda_{1p}\right)-\left(1-\lambda_{1}p\right){log}_{b}\left(1-\lambda_{1}p\right)$$

*leading to:*

(19) $$\frac{dH_{ab}\left(p\right)}{dp}=\lambda_{1}\left({log}_{b}\left(\lambda_{1}p\right)-{log}_{b}\left(1-\lambda_{1}p\right)\right)=0$$

*which is satisfied for the same conditions as above, that is, if* $\lambda_{1}p*=1-\lambda_{1}p*=1/2$ *so that* $p*=1/2\lambda_{1}$ *again. However,* $H_{ab}\left(p*\right)\neq H_{a}\left(p*\right)$ *if* b≠e *(*e *being the basis of the neperian algorithms).*

**Example** **3.** *Consider the particular case of Example 2 for* n=2 *with global deflated probability, that is,* $p_{1}=\lambda_{1}p$ *and* $p_{2}=\lambda_{2}p<1-\lambda_{1}p$ *. The system of two events* $\left\{A_{1},\;A_{2}\right\}$ *is not complete and we extend it with a new event* $A_{3}$ *of positive probability* $p_{3}=1-\left(\lambda_{1}+\lambda_{2}\right)p$ *so that the extended system of events is complete. Now, one has for such an extended system:*

(20) $${\hat{H}}_{a}\left(p\right)=-\lambda_{1}pln\left(\lambda_{1}p\right)-\lambda_{2}pln\left(\lambda_{2}p\right)-\left(1-\left(\lambda_{1}+\lambda_{2}\right)p\right)ln\left(1-\left(\lambda_{1}+\lambda_{2}\right)p\right)$$

(21) $$\begin{array}{c} \frac{d{\hat{H}}_{a}\left(p\right)}{dp}=-\lambda_{1}ln\left(\lambda_{1}p\right)-\lambda_{1}p\frac{\lambda_{1}}{\lambda_{1}p}-\lambda_{2}ln\left(\lambda_{2}p\right)-\lambda_{2}p\frac{\lambda_{2}}{\lambda_{1}p}\\ +\left(\lambda_{1}+\lambda_{2}\right)ln\left(1-\left(\lambda_{1}+\lambda_{2}\right)p\right)-\left(1-\left(\lambda_{1}+\lambda_{2}\right)p\right)\frac{-\left(\lambda_{1}+\lambda_{2}\right)}{1-\left(\lambda_{1}+\lambda_{2}\right)p}\\ =-\lambda_{1}ln\left(\lambda_{1}p\right)-\lambda_{1}-\lambda_{2}ln\left(\lambda_{2}p\right)-\lambda_{2}+\left(\lambda_{1}+\lambda_{2}\right)ln\left(1-\left(\lambda_{1}+\lambda_{2}\right)p\right)+\lambda_{1}+\lambda_{2}\\ =-\lambda_{1}ln\left(\lambda_{1}p\right)-\lambda_{2}ln\left(\lambda_{2}p\right)+\left(\lambda_{1}+\lambda_{2}\right)ln\left(1-\left(\lambda_{1}+\lambda_{2}\right)p\right)\\ =ln\;\frac{{\left(1-\left(\lambda_{1}+\lambda_{2}\right)p\right)}^{\lambda_{1}+\lambda_{2}}}{{\left(\lambda_{1}p\right)}^{\lambda_{1}p}{\left(\lambda_{2}p\right)}^{\lambda_{2}p}}=0 \end{array}$$

*which is satisfied for* p=p* *such that* $\frac{{\left(1-\left(\lambda_{1}+\lambda_{2}\right)p*\right)}^{\lambda_{1}+\lambda_{2}}}{{\left(\lambda_{1}p*\right)}^{\lambda_{1}p*}{\left(\lambda_{2}p*\right)}^{\lambda_{2}p*}}=1$ *. Note that* ${\hat{H}}_{a}\left(p\right)>H_{a}\left(p\right)$ *with* $H_{a}\left(p\right)$ *being obtained in Example 2 for the non-deflated probability case, as expected from Theorem 2. Note also that the value of* p* *giving the maximum entropy is less than the one producing the maximum entropy in the non-deflated case of Example 2 since that one* $p*=1/\left(\lambda_{1}+\lambda_{2}\right)$ *with give the contradiction 0=1 in the above formula for* p=p* *and* $p*<1/\left(\lambda_{1}+\lambda_{2}\right)$ *would give the contradiction* 1<0 *in such a mentioned formula.*

The next result relies on the case where the probabilities are uncertain but each nominal probability is assumed to be eventually subject to the whole set of probabilistic uncertainties so as to cover a wider class of potential probabilistic uncertainties. It is also assumed (contrarily to the assumptions of Theorem 1 and Theorem 2) that the probability disturbances never increase each particular probability of the nominal system of event since they are all constrained to the real interval [0, 1]. As a result, the cardinalities of the extended current and nominal systems of events are, in general, distinct. It is found that the entropy of the current system of events is non-smaller than the nominal entropy.

**Theorem** **3.** *Let* ${\bar{H}}_{\varepsilon \delta }=H\left(p_{1}\delta_{1},p_{1}\delta_{2},\ldots ..,p_{1}\delta_{m},\;\ldots .,\;p_{n}\delta_{1},p_{n}\delta_{2},\ldots ..,p_{n}\delta_{m}\right)$ *and* $H_{*}=H\left(p_{1},p_{2},\ldots ..,p_{n}\right)$ *the extended perturbed and current entropies of the sets of nonempty events* $A_{\alpha }=\left\{A_{ij}:\left(i,j\right)\in \bar{n}\times \bar{m}\right\}$ *and* $A_{*}=\left\{A_{i}:i\in \bar{n}\right\}$ *, respectively, of respective cardinalities* n×m *and* n *, with* $p_{i},\delta_{i}\in \left[0,\;1\right]$ *and* $\sum_{i=1}^{n}p_{i}=\sum_{i=1}^{m}\delta_{i}=1$ *. Then, the following properties hold:*

***(i)***  ${\bar{H}}_{\varepsilon \delta }=H_{*}+{\tilde{H}}_{\delta }\geq H_{*}$ *, where* ${\tilde{H}}_{\delta }=H\left(\delta_{1},\delta_{2},\ldots ..,\delta_{m}\right)$ *is the incremental entropy due to the uncertainties and* ${\bar{H}}_{\varepsilon \delta }=H_{*}$ *if and only if* $\delta_{j}=1$ *and* $\delta_{i}=0$ *;* $\forall i\left(\neq j\right)\in \bar{m}$ *and some arbitrary* $j\in \bar{m}$ *.*
*If* m=n *and* $\delta_{i}=1+\varepsilon_{i}$ *with* $\varepsilon_{i}\in \left[0,\;1\right]$ *;* $\forall i\in \bar{n}$ *and* $\sum_{i=1}^{n}\varepsilon_{i}=1-n$ *then the above relations hold with* ${\bar{H}}_{\varepsilon \delta }=H_{*}$ *if and only if* $\varepsilon_{j}=0$ *and* $\varepsilon_{i}=-1$ *;* $\forall i\left(\neq j\right)\in \bar{n}$ *and some arbitrary* $j\in \bar{n}$ *.*
***(ii)***  *Assume that* m=n *and* $\delta_{i}=1-\varepsilon_{i}$ *with* $\varepsilon_{i}\in \left[-1,\;0\right]$ *;* $\forall i\in \bar{n}$ *and* $\sum_{i=1}^{n}\varepsilon_{i}=n-1$ *. Then,*
  (22) $$\begin{array}{c} {\tilde{H}}_{1-\varepsilon }=H\left(n-k+\sum_{i=1}^{n-k}\varepsilon_{i},\;1+\varepsilon_{k+1},\ldots .,\;1+\varepsilon_{n}\right)+\\ \sum_{j=1}^{n-k}\left(\sum_{i=1}^{j}\varepsilon_{i}+j\right)H\left(\frac{\sum_{i=1}^{n-k-1}\varepsilon_{i}+n-2}{\sum_{i=1}^{n-k}\varepsilon_{i}+n-1},\;\frac{\varepsilon_{n-k}+1}{\sum_{i=1}^{n-k}\varepsilon_{i}+n-1}\right) \end{array}$$
  *for any given integer* n≥3 *and any* $k\in \bar{n-2}$ *such that* $0<n-k+\sum_{i=1}^{n-1}\varepsilon_{i}<1$ *.*
***(iii)***  *The following identity holds under the constraints of Property (ii):*
  (23) $$\begin{array}{c} \sum_{j=1}^{n-k}\left(\sum_{i=1}^{j}\varepsilon_{i}+j\right)H\left(\frac{\sum_{i=1}^{n-k-1}\varepsilon_{i}+n-2}{\sum_{i=1}^{n-k}\varepsilon_{i}+n-1},\;\frac{\varepsilon_{n-k}+1}{\sum_{i=1}^{n-k}\varepsilon_{i}+n-1}\right)\\ ={\tilde{H}}_{\delta }-H\left(n-k+\sum_{i=1}^{n-k}\varepsilon_{i},\;1+\varepsilon_{k+1},\ldots .,\;1+\varepsilon_{n}\right)\geq 0 \end{array}$$
  *for any given integer* n≥3 *and any* $k\in \bar{n-2}$ *such that* $0<n-k+\sum_{i=1}^{n-1}\varepsilon_{i}<1$ *.*

**Proof:**  Note from the additive property of the entropy that ${\bar{H}}_{\varepsilon \delta }=H_{*}+{\tilde{H}}_{\delta }$ and $p_{i},\delta_{j}\in \left[0,\;1\right]$; $\forall i\in \bar{n}$, $\forall j\in \bar{m}$, $\sum_{i=1}^{m}\delta_{i}=1$, equivalently, $\varepsilon_{i}\left(=\delta_{i}-1\right)\in \left[-1,\;0\right]$ and $\sum_{i=1}^{n}\varepsilon_{i}=n-1$ if m=n. Then, Property (i) follows from the non-negativity property of the entropy and the fact that ${\tilde{H}}_{\delta }=0$ if and only if $\delta_{j}=1$ and $\delta_{i}=0$; $\forall i\left(\neq j\right)\in \bar{m}$ and some arbitrary $j\in \bar{m}$ what implies that $H\left(\delta_{1},\delta_{2},\ldots ..,\delta_{m}\right)=H\left(0,0,\ldots ,\delta_{j}\;\left(=1\right),0,\ldots ..,0\right)=0$. The property for m=n and $\varepsilon_{i}=\delta_{i}-1$; $\forall i\in \bar{n}$ is a particular case of the above one. Property (i) has been proved.

On the other hand, one gets from the recursive property of the entropy if m=n and $\varepsilon_{i}=\delta_{i}-1$; $\forall i\in \bar{n}$ with $p_{i}\in \left[0,\;1\right]$, $\varepsilon_{i}\in \left[-1,\;0\right]$; $\forall i\in \bar{n}$, $\sum_{i=1}^{n}p_{i}=1$ and $\sum_{i=1}^{n}\varepsilon_{i}=1-n$ that:

(24) $$\begin{array}{c} {\tilde{H}}_{1-\varepsilon }=H\left(2+\varepsilon_{1}+\varepsilon_{2},\;1+\varepsilon_{3},\;\ldots .,\;1+\varepsilon_{n}\right)+\left(2+\varepsilon_{1}+\varepsilon_{2}\right)H\left(\frac{1+\varepsilon_{1}}{2+\varepsilon_{1}+\varepsilon_{2}},\;\frac{1+\varepsilon_{2}}{2+\varepsilon_{1}+\varepsilon_{2}}\right)\\ =H\left(3+\varepsilon_{1}+\varepsilon_{2}+\varepsilon_{3},\;1+\varepsilon_{4},\;\ldots .,\;1+\varepsilon_{n}\right)\\ +\left(3+\varepsilon_{1}+\varepsilon_{2}+\varepsilon_{3}\right)H\left(\frac{2+\varepsilon_{1}+\varepsilon_{2}}{3+\varepsilon_{1}+\varepsilon_{2}+\varepsilon_{3}},\;\frac{1+\varepsilon_{3}}{3+\varepsilon_{1}+\varepsilon_{2}+\varepsilon_{3}}\right)\\ +\left(2+\varepsilon_{1}+\varepsilon_{2}\right)H\left(\frac{1+\varepsilon_{1}}{2+\varepsilon_{1}+\varepsilon_{2}},\;\frac{1+\varepsilon_{2}}{2+\varepsilon_{1}+\varepsilon_{2}}\right)\\ =H\left(3+\varepsilon_{1}+\varepsilon_{2}+\varepsilon_{3},\;1+\varepsilon_{4},\;\ldots .,\;1+\varepsilon_{n}\right)+\sum_{j=1}^{3}\left(\sum_{i=1}^{j}\varepsilon_{i}+3\right)H\left(\frac{\sum_{i=1}^{2}\varepsilon_{i}+2}{\sum_{i=1}^{3}\varepsilon_{i}+3},\;\frac{\varepsilon_{3}+1}{\sum_{i=1}^{3}\varepsilon_{i}+3}\right)\\ =H\left(3+\varepsilon_{1}+\varepsilon_{2}+\varepsilon_{3},\;1+\varepsilon_{4},\;\ldots .,\;1+\varepsilon_{n}\right)+\sum_{j=1}^{3}\left(\sum_{i=1}^{j}\varepsilon_{i}+3\right)H\left(\frac{\sum_{i=1}^{2}\varepsilon_{i}+2}{\sum_{i=1}^{3}\varepsilon_{i}+3},\;\frac{\varepsilon_{3}+1}{\sum_{i=1}^{3}\varepsilon_{i}+3}\right)\\ =H\left(n-k+\sum_{i=1}^{n-k}\varepsilon_{i},\;1+\varepsilon_{k+1},\ldots .,\;1+\varepsilon_{n}\right)+\\ \sum_{j=1}^{n-k}\left(\sum_{i=1}^{j}\varepsilon_{i}+j\right)H\left(\frac{\sum_{i=1}^{n-k-1}\varepsilon_{i}+n-2}{\sum_{i=1}^{n-k}\varepsilon_{i}+n-1},\;\frac{\varepsilon_{n-k}+1}{\sum_{i=1}^{n-k}\varepsilon_{i}+n-1}\right)\\ =H\left(n-1+\sum_{i=1}^{n-1}\varepsilon_{i},\;1+\varepsilon_{n}\right)+\sum_{j=1}^{n-1}\left(\sum_{i=1}^{j}\varepsilon_{i}+j\right)H\left(\frac{\sum_{i=1}^{n-2}\varepsilon_{i}+n-2}{\sum_{i=1}^{n-1}\varepsilon_{i}+n-1},\;\frac{\varepsilon_{n-1}+1}{\sum_{i=1}^{n-1}\varepsilon_{i}+n-1}\right) \end{array}$$

for any given integer n≥3 each equality for a right-hand-side $H\left(n-k+\left(\ldots \right),\ldots .,,1+\varepsilon_{n}\right)$ is valid for each $k\in \bar{n-2}$ such that $p_{i}\in \left[0,\;1\right]$, $\varepsilon_{i}\in \left[-1,\frac{1-p_{i}}{p_{i}}\right]$; $\forall i\in \bar{n}$, $0<n-k+\sum_{i=1}^{n-1}\varepsilon_{i}<1$ implying that the sum of the two first argument of the entropy is positive, that is, $\varepsilon_{1}+\varepsilon_{2}>-2$. Property (ii) has been proved. Property (iii) is a consequence of Property (ii). □

The next result relies on the case where the probabilities are uncertain but belonging to a known admissibility real interval, rather than fixed as it has been assumed in Lemma 1. Contrarily to Theorem 3, it is not assumed that the probabilistic disturbances are within [0, 1] so that they can increase the corresponding nominal probabilities.

**Theorem** **4.** *Assume a finite complete system of events* $\left\{A_{i}:i\in \bar{n}\right\}$ *such that the nominal and current probabilities of the event* $A_{i}$ *are* $p_{i}$ *and* $p_{i}\left(1+\varepsilon_{i}\right)$ *, respectively, with* $\varepsilon_{i}\in \left[-\varepsilon_{i0},\;\varepsilon_{i1}\right]$ *, such that* $\varepsilon_{i1}+\varepsilon_{i0}\geq 0$ *(so that the constraint* $1+\varepsilon_{i1}\geq 1-\varepsilon_{i0}$ *holds,* $\varepsilon_{i0}\in \left[\left(p_{i}-1\right)/p_{i},\;1\right]$ *and* $-1\leq \varepsilon_{i1}\leq \left(1-p_{i}\right)/p_{i}$ *;* $\forall i\in \bar{n}$ *and* $\sum_{i=1}^{n}p_{i}\left(1+\varepsilon_{i}\right)=1$ *. Then, the following properties hold:*

***(i)***  $H_{\varepsilon }\geq max\;\left[\left(1+{\bar{\varepsilon }}_{0}\right)H_{*}-\left(1+{\bar{\varepsilon }}_{1}\right),\;0\right]$ *with* ${\bar{\varepsilon }}_{1}=\underset{1\leq i\leq n}{max}\left|\varepsilon_{i}\right|$ *and* ${\bar{\varepsilon }}_{0}=\underset{1\leq i\leq n}{min}\left|\varepsilon_{i}\right|$
*where* $H_{\varepsilon }=H\left(p_{1}\left(1+\varepsilon_{1}\right),p_{2}\left(1+\varepsilon_{2}\right),\ldots ..,p_{n}\left(1+\varepsilon_{n}\right)\right)$ *and* $H_{*}=H\left(p_{1},p_{2},\ldots ..,p_{n}\right)$
***(ii)***  (25) $$\left(1-m_{0}\right)H_{*}\geq H_{\varepsilon }\geq \left(1+m_{1}\right)H_{*}$$
  *where:*
  (26) $$m_{0}=1-\sum_{i=1}^{n}p_{i}lnp_{i}\left(\sum_{j=0}^{\infty }\left(\frac{1}{2j+1}+\frac{\varepsilon_{i0}}{2\left(j+1\right)}\right){\left(-\varepsilon_{i0}\right)}^{2j+1}\left(1+\varepsilon_{i1}\right)\right)$$
  (27) $$m_{1}=\sum_{j=0}^{\infty }\left(\frac{1}{2j+1}-\frac{\varepsilon_{i1}}{2\left(j+1\right)}\right)\varepsilon_{i1}^{2j+1}\left(1+\varepsilon_{i1}\right)-1$$
***(iii)***  *If* $\varepsilon_{i0}\in \left[\left(p_{i}-1\right)/p_{i},\;1\right)$ *and* $\varepsilon_{i1}\in \;\left(-1,\;\left(1-p_{i}\right)/p_{i}\right]$ *then:*
  (28) $$\sum_{i=1}^{n}p_{i}lnp_{i}\left(1-\varepsilon_{i0}\right)\frac{\varepsilon_{i0}}{\left|1-\varepsilon_{i0}\right|}\geq H_{\varepsilon }\geq -\sum_{i=1}^{n}p_{i}lnp_{i}\left(1+\varepsilon_{i1}\right)\varepsilon_{i1}$$
***(iv)***  *If* $\varepsilon_{i0}\in \left[\left(p_{i}-1\right)/p_{i},\;1\right)$ *and* $\varepsilon_{i1}\in \;\left(-1,\;\left(1-p_{i}\right)/p_{i}\right]$ *then:*
  (29) $$-\sum_{i=1}^{n}p_{i}lnp_{i}\left(1+\varepsilon_{i1}\right)\varepsilon_{i1}=H_{0}\leq H_{\varepsilon }\leq {\bar{H}}_{1}={\left(\sum_{i=1}^{n}\varepsilon_{i0}^{2}\right)}^{1/2}H\left(p_{1},p_{2},\ldots ..p_{n}\right)$$
  *such that an upper-bound of the lower-bound* $H_{0}$ *of* $H_{\varepsilon }$ *is:*
  (30) $${\bar{H}}_{0}=H_{*}{\left(\sum_{i=1}^{n}{\left(1+\varepsilon_{i1}\right)}^{2}\varepsilon_{i1}^{2}\right)}^{1/2}$$

**Proof:**  Note that, since $\varepsilon_{i}>-1$; $\forall i\in \bar{n}$ then $ln\left(1+\varepsilon_{i}\right)\leq \varepsilon_{i}$; $\forall i\in \bar{n}$ so that:

(31) $$\begin{array}{c} -H_{\varepsilon }=\sum_{i=1}^{n}p_{i}\left(1+\varepsilon_{i}\right)\left(lnp_{i}+ln\left(1+\varepsilon_{i}\right)\right)\\ \leq \sum_{i=1}^{n}p_{i}\left(1+\varepsilon_{i1}\right)\left(\varepsilon_{i1}+lnp_{i}\right)\\ \leq -H_{*}+\sum_{i=1}^{n}p_{i}lnp_{i}\varepsilon_{i}+\sum_{i=1}^{n}p_{i}\left(1+\varepsilon_{i}\right)\varepsilon_{i}\\ \leq -H_{*}-{\bar{\varepsilon }}_{0}H_{*}+\left(1+{\bar{\varepsilon }}_{1}\right)\sum_{i=1}^{n}p_{i}\varepsilon_{i}\\ =-H_{*}-{\bar{\varepsilon }}_{0}H_{*}+\left(1+{\bar{\varepsilon }}_{1}\right)\\ =-\left(1+{\bar{\varepsilon }}_{0}\right)H_{*}+\left(1+{\bar{\varepsilon }}_{1}\right) \end{array}$$

and Property (i) follows directly. On the other hand, note that:

(32) $$\begin{array}{c} \sum_{i=1}^{n}p_{i}\left(1-\varepsilon_{i0}\right)\left(lnp_{i}+ln\left(1-\varepsilon_{i0}\right)\right)\\ \leq -H_{*}\leq \sum_{i=1}^{n}p_{i}\left(1+\varepsilon_{i}\right)\left(lnp_{i}+ln\left(1+\varepsilon_{i}\right)\right) \end{array}$$

Note that, since $max\left(\varepsilon_{i0},\;\varepsilon_{i1}\right)\leq 1$; $\forall i\in \bar{n}$, we can use Taylor’s expansion series around 1 for the above neperian logarithms to get:

(33) $$\begin{array}{c} ln\left(1+\varepsilon_{i1}\right)=\sum_{j=0}^{\infty }\frac{{\left(-1\right)}^{n}\varepsilon_{i1}^{j+1}}{j+1}=\sum_{j=0}^{\infty }\frac{\varepsilon_{i1}^{2j+1}}{2j+1}-\sum_{j=0}^{\infty }\frac{\varepsilon_{i1}^{2\left(j+1\right)}}{2\left(j+1\right)}\\ =\sum_{j=0}^{\infty }\left(\frac{\varepsilon_{i1}^{2j+1}}{2j+1}-\frac{\varepsilon_{i1}^{2\left(j+1\right)}}{2\left(j+1\right)}\right)=\sum_{j=0}^{\infty }\left(\frac{1}{2j+1}-\frac{\varepsilon_{i1}}{2\left(j+1\right)}\right)\varepsilon_{i1}^{2j+1} \end{array}$$

(34) $$ln\left(1-\varepsilon_{i0}\right)=\sum_{j=0}^{\infty }\frac{{\left(-1\right)}^{j}{\left(-\varepsilon_{i0}\right)}^{j+1}}{j+1}=\sum_{j=0}^{\infty }\left(\frac{1}{2j+1}+\frac{\varepsilon_{i0}}{2\left(j+1\right)}\right){\left(-\varepsilon_{i0}\right)}^{2j+1}$$

so that:

(35) $$\begin{array}{c} \sum_{i=1}^{n}p_{i}lnp_{i}\left(1-m_{0}\right)=-\left(1-m_{0}\right)H_{*}=-H_{1}\\ \leq -H_{\varepsilon }\\ \leq -H_{0}=-\left(1+m_{1}\right)H_{*}=\sum_{i=1}^{n}p_{i}lnp_{i}\left(1+m_{1}\right) \end{array}$$

(36) $$\ln\left(1+\varepsilon_{i1}\right)=\sum_{j=0}^{\infty }\frac{{\left(-1\right)}^{n}\varepsilon_{i1}^{j+1}}{j+1}=\sum_{j=0}^{\infty }\frac{\varepsilon_{i1}^{2j+1}}{2j+1}-\sum_{j=0}^{\infty }\frac{\varepsilon_{i1}^{2\left(j+1\right)}}{2\left(j+1\right)}$$

so that $H_{1}\geq H_{*}\geq H_{0}$. Since $\varepsilon_{i1}\underset{j\geq 0}{min}\frac{2j+2}{2j+1}=\underset{j\to \infty }{lim}\frac{2j+2}{2j+1}=1$ then $m_{1}\geq -1$ so that $H_{0}\geq 0$ and $H_{1}\geq H_{0}\geq 0$. Furthermore, (26) and (27) imply that $m_{1}\in \left[-1,\;0\right]$ and $m_{0}\in \left[0,\;1\right]$. The proof of Property (i) has been completed. Now, since $\varepsilon_{i0}<1$ and $\varepsilon_{i1}>-1$; $\forall i\in \bar{n}$ then (25) follows since:

(37) $$ln\left(1+\varepsilon_{i}\right)\leq ln\left(1+\varepsilon_{i1}\right)\leq \varepsilon_{i1}$$

since $\varepsilon_{i1}>-1$; $\forall i\in \bar{n}$:

(38) $$ln\left(1+\varepsilon_{i}\right)\geq ln\left(1-\varepsilon_{i0}\right)\geq -\frac{\varepsilon_{i0}}{1-\varepsilon_{i0}}=\frac{\varepsilon_{i0}}{\varepsilon_{i0}-1}$$

since $-\varepsilon_{i0}>-1$; $\forall i\in \bar{n}$, equivalently, $\varepsilon_{i0}<1$; $\forall i\in \bar{n}$ so that:

(39) $$\begin{array}{c} -\sum_{i=1}^{n}p_{i}lnp_{i}\left(1-\varepsilon_{i0}\right)\frac{\varepsilon_{i0}}{\left|1-\varepsilon_{i0}\right|}=\sum_{i=1}^{n}p_{i}lnp_{i}sgn\left(1-\varepsilon_{i0}\right)\varepsilon_{i0}\\ \leq \sum_{i=1}^{n}p_{i}lnp_{i}\left(1-\varepsilon_{i0}\right)ln\left(1-\varepsilon_{i0}\right)\\ \leq -H\left(p_{1}\left(1+\varepsilon_{1}\right),p_{2}\left(1+\varepsilon_{2}\right),\ldots ..p_{n}\left(1+\varepsilon_{n}\right)\right)=\sum_{i=1}^{n}p_{i}lnp_{i}\left(1+\varepsilon_{i}\right)ln\left(1+\varepsilon_{i}\right)\\ \leq \sum_{i=1}^{n}p_{i}lnp_{i}\left(1+\varepsilon_{i1}\right)ln\left(1+\varepsilon_{i1}\right)\\ \leq \sum_{i=1}^{n}p_{i}lnp_{i}\left(1+\varepsilon_{i1}\right)\varepsilon_{i1} \end{array}$$

and Property (ii) has been proved. On the other hand, Property (iii) is proved as follows:

(40) $$\begin{array}{c} H_{\varepsilon }=-\sum_{i=1}^{n}p_{i}lnp_{i}\left(1+\varepsilon_{i}\right)ln\left(1+\varepsilon_{i}\right)=\sum_{i=1}^{n}p_{i}lnp_{i}\frac{\left(1-\varepsilon_{i0}\right)\varepsilon_{i0}}{\left|1-\varepsilon_{i0}\right|}\\ \sum_{i=1}^{n}p_{i}lnp_{i}\left(1-{\bar{\varepsilon }}_{i0}\right)\frac{\varepsilon_{i0}}{\left|1-\varepsilon_{i0}\right|}=\sum_{i=1}^{n}p_{i}lnp_{i}sign\left(1-\varepsilon_{i0}\right)\varepsilon_{i0}\\ \leq {\left(\sum_{i=1}^{n}{\left(sign\left(1-\varepsilon_{i0}\right)\varepsilon_{i0}\right)}^{2}\right)}^{1/2}{\left(\sum_{i=1}^{n}{\left(p_{i}lnp_{i}\right)}^{2}\right)}^{1/2}\\ \leq {\left(\sum_{i=1}^{n}\varepsilon_{i0}^{2}\right)}^{1/2}\left|H_{*}\right| \end{array}$$

After using of Cauchy-Schwartz inequality for summable sequences and the constraint ${\left(\sum_{i=1}^{n}{\left(p_{i}lnp_{i}\right)}^{2}\right)}^{1/2}\leq H_{*}$, one gets, since $\varepsilon_{i1}>-1$ and $\varepsilon_{i0}<1$, that Property (iii) holds since:

(41) $$\begin{array}{c} -\sum_{i=1}^{n}p_{i}lnp_{i}\left(1+\varepsilon_{i1}\right)ln\left(1+\varepsilon_{i}\right)=-\sum_{i=1}^{n}p_{i}lnp_{i}\left(1+\varepsilon_{i}\right)ln\left(1+\varepsilon_{i}\right)\frac{\left(1+\varepsilon_{i1}\right)\varepsilon_{i1}}{\left(1+\varepsilon_{i}\right)ln\left(1+\varepsilon_{i}\right)}\\ \leq {\left(\sum_{i=1}^{n}\frac{{\left(1+\varepsilon_{i1}\right)}^{2}\varepsilon_{i1}^{2}}{{\left(1+\varepsilon_{i}\right)}^{2}{ln}^{2}\left(1+\varepsilon_{i}\right)}\right)}^{1/2}H\left(p_{1},p_{2},\ldots ..p_{n}\right)\\ \leq {\left(\sum_{i=1}^{n}\frac{{\left(1+\varepsilon_{i1}\right)}^{2}\varepsilon_{i1}^{2}}{{\left(1-\varepsilon_{i0}\right)}^{2}{ln}^{2}\left(1-\varepsilon_{i0}\right)}\right)}^{1/2}H\left(p_{1},p_{2},\ldots ..p_{n}\right) \end{array}$$

and Property (iv) follows from Property (iii) and the fact that:

(42) $$\begin{array}{c} H_{0}=-\sum_{i=1}^{n}p_{i}lnp_{i}\left(1+\varepsilon_{i1}\right)\varepsilon_{i1}\\ \leq {\left(\sum_{i=1}^{n}\left(p_{i}^{2}{ln}^{2}p_{i}\right)\right)}^{1/2}{\left(\sum_{i=1}^{n}{\left(1+\varepsilon_{i1}\right)}^{2}\varepsilon_{i1}^{2}\right)}^{1/2}\leq {\bar{H}}_{0}={\left(\sum_{i=1}^{n}{\left(1+\varepsilon_{i1}\right)}^{2}\varepsilon_{i1}^{2}\right)}^{1/2}H_{*} \end{array}$$

□

## 4. Applications to Epidemic Systems

This section is devoted to the application of entropy tools to the modelling of epidemics. It should be pointed first out that some parameters of the epidemic description evolutions, typically the coefficient transmission rate, can vary according to seasonality [30]. It can be also pointed out that the existing medical tests to evaluate the proportions of healthy and infected individuals are not always subject to confluent estimation of the errors. That is the estimated worst-case errors for reach of the populations can have different running ranges due to the fact that they are performed with different techniques. See, for instance, [31,32] where the mentioned question is justified taking as a basis medical tests.

-To solve the first problem, one considers the usefulness of designing a parallel scheme of alternative time-invariant models, being ran by a supervisory switching law, to choose the most appropriate active time-invariant model through time. The whole set of models covers the whole range of expected variations of the model parameters through time. The objective of the parallel structure is to select the one which describes the registered data more tightly along a certain period of time.

-On the other hand, the fact that the existing tests of errors on the subpopulation integrating the model not always give similar worst-case allowed estimated errors for all the subpopulations, justifies and adjustment of the probabilities in the case when the sum of all of them does not equalize unity. Two potential actions to overcome this drawback are: (a) the introduction of events of positive (respectively, negative) probabilities in the case of deflated (respectively, inflated) global probability; (b) to readjust all the individuals estimated probabilities via normalization by the current sum which is distinct of unity. In the first case, the re-adjustment is made by an algebraic sum manipulation. In the second one, be readjusting via normalization all the individual probabilities. In both cases, it is achieved that the amended global probability is unity.

Assume an epidemic disease with unknown time-varying bounded coefficient transmission rate $\beta \left(t\right)\in \left[\beta^{0},\;\beta^{1}\right]$, where $\beta^{0}$ and $\beta^{1}$ are known, which is defined by the following differential system of n first-order differential equations:

(43) $$\dot{x}\left(t\right)=\Psi \left(x\left(t\right),\beta \left(t\right)\;,p\right)x\left(t\right)+\Gamma u\left(t\right);\;x\left(0\right)=x_{0}$$

where $x\left(t\right)\in R^{n}$ is the state-vector and p is the vector of parameters containing all other parameters that the coefficient transmission rate, like recovery rate mortality rate, average survival rate, average expectation of life irrespective of the illness etc. In practice, Equation (43) can be replaced by a non-parameterized description, based on the state measurements through time, where x(t) is given by provided experimental on-line data on the subpopulation which in this case, should be discretized with a small sampling period. In this way, (43) can be either a more sophisticated mathematical model, than those simplified ones provided later on, which provides data x(t) on the illness close to the real measurements or the listed real data themselves got from the disease evolution. The state vector contains the subpopulations integrated in the model which depend on the type of model itself such as susceptible (E), infectious (I) and recovered (or immune) (R) in the so-called SIR models to which it is added, in the so-called SEIR models the exposed subpopulation (E) which are those in the first infection stages with no external symptoms. The models can also contain a vaccinated subpopulation (V) and can have also several nodes or patches, describing, for instance, different environments, in general coupled, each having their own set of coupled subpopulations which interact with the remaining ones though population fluxes. The vector $u\left(t\right)\in R^{m}$ is the control vector. There are typically either one control, namely, vaccination on the susceptible, or two controls, namely, vaccination on the susceptible and (either antiviral or antibiotic) treatment on the infectious in the case when there is only one node. Those controls might be applied to each subsystem associated to one patch if there are several patches integrated in the model. The matrix function of dynamics Ψ(x(t),β(t) ,p) is a real n×n-matrix for each $t\in R_{0+}$. The control matrix Γ has as many columns as controls are applied and it typically consists of entries being “o” (i.e., no control applied on the corresponding state component associated to one subpopulation), “−1” if the control leads to a decrease of the rate of growing of a subpopulation, for instance, vaccination effort on the susceptible) and “+1” if it leads to a compensatory increase rate of a subpopulation due to a corresponding decrease of another one, for instance, the increase in the recovered in the vaccination case (when the susceptible are decreased via vaccination) or again the recovered in the treatment case (when the infectious are decreased via treatment). For simplicity, it is assumed that p is constant and there are no delays in the dynamics.

*The control architecture which is proposed consists of a scheme of approximated models located in a parallel disposal, one of them being chosen by a higher- level supervisory switching scheme as the active model to select the controller gains along each current time interval*.

In particular, it is proposed to run a set of Q+1 approximated models of the same dimension as Equation (43) with a constant coefficient transmission rate $\beta_{i}$; $\forall i\in \bar{Q+1}$ being chosen as:

(44) $${\dot{x}}_{i}\left(t\right)=\Psi_{i}\left(x_{i}\left(t\right),\beta_{i}\;,p_{i}\right)x\left(t\right)+\Gamma u_{i}\left(t\right);\;x_{i}\left(0\right)=x_{0},\;\forall i\in \bar{Q+1}$$

with $x_{i}\left(t\right)\in R^{n}$,where:

(45) $$\beta_{i+1}=\beta_{i}+\frac{\beta^{1}-\beta^{0}}{Q};\;\forall i\left(>1\right)\in \bar{Q},\;\beta_{1}=\beta^{0}$$

in such way that $\beta_{1}=\beta^{0}$ and $\beta_{Q+1}=\beta^{1}$. Note that the approximated models (44) and (45) are parameterized by a constant coefficient transmission rate contrarily to the real model (43) whose coefficient transmission rate is time-varying. The remaining parameters are constant and eventually time- varying, respectively. Note also that the models (44) are initialized to the initial conditions. We consider a set of (Q+1) event errors $E=\left\{E_{i}:\;i\in \bar{Q+1}\right\}$ of the states of the models (44) and (45) with respect to (43), that is, $e_{i}\left(t\right)=x_{i}\left(t\right)-x\left(t\right)$; $\forall i\in \bar{Q+1}$. Each event $E_{i}$ is integrated by a set of events $E_{ij}$ which are the errors of each of its integrating subpopulations with respect to the real system, that is, $E_{i}=\left\{E_{ij}\;:j\in \bar{n}\right\}$; $\forall i\in \bar{Q+1}$.

Define the instantaneous error entropies of each error event by summing up all the component-wise contributions, that is, $H\left(E_{i},\;t\right)=-\sum_{j=1}^{n}p_{ij}\left(t\right)lnp_{ij}\left(t\right)$; $\forall i\in \bar{Q+1}$, while the corresponding accumulated continuous-time and discrete-time entropies on the time interval [t,t+T) are defined in a natural way from the instantaneous ones, respectively, as follows:

(46) $$H_{ca}\left(E_{i},\;\left[t,t+T\right]\right)=-\int_{t}^{t+T}H\left(E_{i},t+j\tau \right)d\tau =-\int_{t}^{t+T}\sum_{j=1}^{n}p_{ij}\left(\sigma \right)lnp_{ij}\left(\sigma \right)d\sigma$$

and:

(47) $$H_{da}\left(E_{i},\;\left[t,t+T\right]\right)=-\sum_{j=0}^{\alpha }H\left(E_{i},t+j\tau \right)=-\sum_{k=0}^{\alpha }\sum_{j=1}^{n}p_{ij}\left(t+k\tau \right)lnp_{ij}\left(t+k\tau \right)$$

provided that T=ατ so that α=T/τ is the set of sampling intervals on T of period τ which is a submultiple of T with T and τ being design parameters satisfying these constraints.

The control effort is calculated by applying on a time interval $\left[t,t_{i+1}\right)$ the control which has made the accumulated entropy of the error on an error event to be smallest one among all the error events on a tested previous time interval $\left[t-t_{i},\;t\right)$ which defines the so-called active model on $\left[t,\;t_{i+1}\right)$. To simplify the exposition, and with no loss in generality, the accumulated discrete - time entropy is the particular one used for testing in the sequel. Then, the following switching Algorithm 1 is proposed:
**Algorithm 1** (all the subpopulations are ran by the same active model within each inter-switching interval)  **Step 0**- Auxiliary design parameters:  Define the prefixed minimum inter-sample period threshold $T_{min}>0$ and σ being an auxiliary time interval, $0<\sigma <<T_{min}$, to measure the possible degradation of the current active model in operation what foresees a new coming switching.  **Step 1**-Initial control:  $u\left(t\right)=u_{i}\left(t\right)$ for $t=t_{0}$, with $t_{0}=0$, for some arbitrary model $i\in \bar{Q+1}$ and make $k\left(\in Z_{0+}\right)=0$, the initial active control being $a\left(0\right)=i\in \bar{Q+1}$ an the initial running integer for switching time instants is k=0.  **Step 2**- Eventually switched control:
(48)$$\begin{array}{c} u\left(t\right)=\left\{u_{i}\left(t\right):\;H_{da}\left(E_{i},\;\left[t_{k},t\right)\right)=\underset{1\leq j\leq Q+1}{min}H_{da}\left(E_{j},\;\left[t_{k},t\right)\right)\right\};\;\forall t\in \left[t_{k},\;t_{k}+T_{k}\right)\\ \wedge \left(H_{da}\left(E_{i},\;\left[t_{k},t_{k}+\tau \right)\right)>\underset{1\leq j\left(\neq i\right)\leq Q+1}{min}H_{da}\left(E_{j},\;\left[t_{k},t_{k}+\tau \right)\right)\right)\} \end{array}$$such that:  *-current active model*: $a\left(t\right)=i\in \bar{Q+1}$; $\forall t\in \left[t_{k},\;t_{k+1}\right)$ is the active model in the set of (Q+1) models which generates the control on $\left[t_{k},\;t_{k+1}\right)$,  - *next active model*: $a\left(t\right)=j\left(\neq i\right)\in \bar{Q+1}$, such that $H_{da}\left(E_{j},\;\left[t_{k+1},t_{k+1}-\sigma \right]\right)<\underset{1\leq \ell \leq Q+1}{min}H_{da}\left(E_{\ell },\;\left[t_{k+1},t_{k+1}-\sigma \right]\right)$ is the next active model to be in operation at the next switching time instant $t=t_{k+1}$.  - The switching time instants $t_{k+1}=t_{k+1}\left(t_{k}\right)=t_{k}+T_{k}$ and the inter-switching time periods $T_{k}=T_{k}\left(t_{k}\right)\geq T_{min}$ for $j\in Z_{0+}$ depend on the set of preceding sampling instants $\left\{t_{0}=0,t_{1}\left(t_{0}\right),\;t_{2}\left(t_{1}\right),\cdots ,\;t_{k}\left(t_{k-1}\right),\;\ldots \right\}$.  - The control law (48) is calculated by computing the accumulated discrete- time entropies (47) with the following probabilistic rule:
(49)$$p_{ij}\left(t+k\tau \right)=1-\;\frac{k_{ij}\left(t\right)\left|e_{ij}\left(t+k\tau \right)\right|}{\sum_{j=1}^{n}\left|e_{ij}\left(t+k\tau \right)\right|};\;\forall i\in \bar{Q+1},\;\forall j\in \bar{n},\;\forall k\in \bar{T/\tau }\cup \left\{0\right\},\;\forall t\in R_{0+}$$
with $k_{ij}\left(t\right)\in \left(0,\;{\bar{k}}_{ij}\left(t\right)\right)$; $\forall i\in \bar{Q+1}$, $\forall j\in \bar{n}$, and  $e_{ij}\left(t\right)=x_{ij}\left(t\right)-x_{j}\left(t\right)$; $\forall i\in \bar{Q+1}$, $\forall j\in \bar{n}$, $\forall t\in R_{0+}$  **Step 3**-Updating the activation of the next active control and inter-switching time interval:  Do k←k+1 with $t_{k+1}\leftarrow t_{k}+T_{k}$ being the next controller switching time instant to the next active model $a\left(t_{k+1}\right)\in \bar{Q+1}$, re-initialize all the models to the measured data, that is, $x_{i}\left(t_{k+1}\right)=x\left(t_{k+1}\right)$; $\forall i\in \bar{Q+1}$ and Go to Step 2.

**Remarks:**  ***(1)** Note that the initial control run on a time interval lasting at least the designed time interval length* $T_{min}$ *. In the case of availability of some “a priori” knowledge about the adequacy of the various models to the epidemic process in the initial stage of the disease, this knowledge can be used to overcome the arbitrariness in the selection of the initial controller.*

***(2)** Note also that if*${\bar{k}}_{ij}\leq \frac{\sum_{j=1}^{n}\left|e_{ij}\left(t+j\tau \right)\right|}{\left|e_{ij}\left(t+j\tau \right)\right|}$*;*$i\in \bar{Q+1}$*,*$\forall j\in \bar{n}$*,*$\forall t\in R_{0+}$*then the global probability of the error event*$E_{i}$*cannot be inflated but it can be deflated. If*${\bar{k}}_{ij}\left(t\right)=n-1$*;*$i\in \bar{Q+1}$*,*$\forall j\in \bar{n}$*,*$\forall t\in R_{0+}$ then such a global probability is neither inflated nor deflated for all time.

$\sum_{j=1}^{n}p_{ij}\left(t+j\tau \right)=\sum_{j=1}^{n}\left(1-\left(n-1\right)\frac{\left|e_{ij}\left(t+j\tau \right)\right|}{\left(\sum_{j=1}^{n}\left|e_{ij}\left(t+j\tau \right)\right|\right)}\right)=n-\left(n-1\right)=1$ *;* $\forall i\in \bar{Q+1}$ *,* $\forall t\in R_{0+}$ *. Note also that the fact that* $k_{ij}\left(t\right)$ *can be time-varying so that the probabilities have a margin for experimental design adjustment relies with the problem statement of probabilities subject to possible errors in the theoretical statements of the above sections.*

*(**3**) Note that in the above algorithm there are zero-probability (although non impossible) events of leading a unique solution like, for instance, the first accumulated entropy equality in (48) of the strict inequality leading the next active controller. If the event a lack of uniqueness be detected one can fix any valid solution (from the set of valid ones) to run or simply to give an uniqueness rule, as for instance, to get as active the nearest indexed model to the last active one active among the set of valid ones.*

*(**4**) Note also that the events* $E_{ij}$ *for* $j\in \bar{n}$ *and each given* $i\in \bar{Q+1}$ *are not mutually independent since they consist of the solutions of all the subpopulations, each given by a single-order differential equation, between the* n-th *differential equation of the* i *-* th *model. The reason is that first-order equations of the differential system are coupled.*

*(**5**) It can be observed that the switching time instants are also resetting times of initial conditions with the real (or more tightly) data provided by the real model (43).*

*(**6**) The use of Equation (49) to calculate the probabilities together with their companion saturation rules for the control gains, is sufficient to evaluate the entropies of Equation (48) in order to implement the algorithm. So, it is no need of introduction of a compensatory event of negative probability to equalize to unity the global probability.*

An alternative to the above switching algorithm consists of defining the error events, one- per subpopulation of the epidemic description (44) versus their real one counterparts in (43). For instance, we can think of choosing the “susceptible event error” with the Q+1 first error components of all the solutions of (44) compared to the first component of (43) and one proceeds so on for the various remaining subpopulations. So, we can chose online the best simplified model (that is, the closest one to the true complex model) for each subpopulation. Note that this can be reasonable *since the approximated models are designed by choosing parameterization of the coefficient transmission rate which sweep a region where the true one is point-wise allocated through time*. *In parallel, each whole set of error events, associated to each of the subpopulations, consists of mutually independent events* since, in this case, the data of all the set of models, including those of the active one, are generated by different differential equations on nonzero measure intervals. In this case, the set of events is $\left\{E_{1},\;E_{2},\cdots ,\;E_{n}\right\}$, such that $E_{j}=\left\{E_{ij}:i\in \bar{Q+1}\right\}$; $\forall j\in \bar{n}$ is associated to the j-th subpopulation, instead of $\left\{E_{1},\;E_{2},\cdots ,\;E_{Q+1}\right\}$ with $E_{i}=\left\{E_{ij}:j\in \bar{n}\right\}$; $\forall i\in \bar{Q+1}$ as associated to one model as it was sated in the former design. Then, we have the following Algorithm 2.
**Algorithm 2** (each subpopulation can be ran by a different active model within each inter-switching interval)  **Step 0**- *Auxiliary design parameters*: it is similar to that of Algorithm 1.  **Step 1*-****Initial control*: it is similar to that of Algorithm 1.  **Step 2**- Eventually switched control:
(50)$$\begin{array}{c} u_{j}\left(t\right)=\left\{u_{ji}\left(t\right):\;H_{da}\left(E_{j},\;\left[t_{k},t\right)\right)=\underset{1\leq i\leq n}{min}H_{da}\left(E_{i},\;\left[t_{k},t\right)\right)\right\};\;\forall t\in \left[t_{k},\;t_{k}+T_{k}\right)\\ \wedge \left(H_{da}\left(E_{j},\;\left[t_{k},t_{k}+\tau \right)\right)>\underset{1\leq i\left(\neq j\right)\leq n}{min}H_{da}\left(E_{j},\;\left[t_{k},t_{k}+\tau \right)\right)\right)\} \end{array}$$such that $u_{j}\left(t\right)$ is the j-th controller component, and one can distinguish:  *- the current active model for the*j-th subpopulation: $a\left(t\right)=i\in \bar{n}$; $\forall t\in \left[t_{k},\;t_{k+1}\right)$ is the active model in the set of n models which generates the control on $\left[t_{k},\;t_{k+1}\right)$,  *- the next active model for the*
j-th subpopulation: $a\left(t\right)=j\left(\neq i\right)\in \bar{n}$, such that $H_{da}\left(E_{j},\;\left[t_{k+1},t_{k+1}-\sigma \right]\right)<\underset{1\leq \ell \leq n}{min}H_{da}\left(E_{\ell },\;\left[t_{k+1},t_{k+1}-\sigma \right]\right)$ is the next active model to be in operation at the next switching time instant $t=t_{k+1}$.  - The switching time instants are obtained in a similar way as in Algorithm 1. - The control law (33) is calculated by computing the accumulated discrete- time entropies (47) with a similar probabilistic rule as that of Algorithm1 with errors $e_{ij}\left(t\right)=x_{ij}\left(t\right)-x_{j}\left(t\right)$; $\forall i\in \bar{Q+1}$, $\forall j\in \bar{n}$, $,\;\forall t\in R_{0+}$  **Step 3**- Updating the activation of the next active control and inter-switching time interval: it is similar to that of Algorithm 1.

**Remark** **6.** *Related to (50) in Algorithm 2 versus (48) in Algorithm 1, note that* $u_{j}\left(t\right)$ *is the* j *-th controller component. Assume that the epidemic model is a SEIR one such that* $x={\left(x_{1},\;x_{2},x_{3},x_{4}\right)}^{T}={\left(S_{-susceptible},\;E_{-exposed},I_{-infectious},\;R_{-immune}\right)}^{T}$ *. The precise meaning of the sentence is that the active vaccination control is got, for instance, from the active controller* $1\in \bar{n}$ *if feedback vaccination control is used as being proportional to the susceptible subpopulation. And the active treatment control is got, for instance, from the active controller* $3\in \bar{n}$ *if feedback treatment control is used being proportional to the infectious subpopulation. If there are no more controllers the remaining controllers would be zeroed for all time. The models for the other components could be omitted from the whole scheme or simply used for information of the estimation of subpopulations since no controls are got from them to be injected to the real population.*

## 5. Simulation Examples

**Figure 1:**
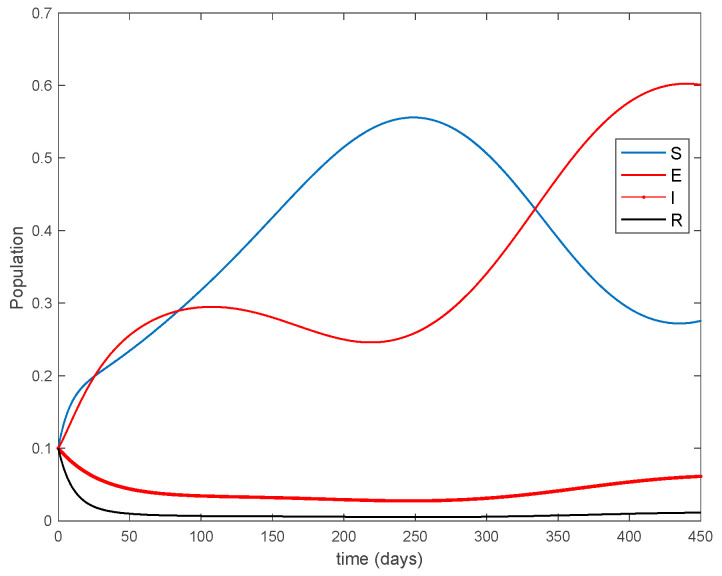
Dynamics of the accurate seasonal epidemic model.

This section contains some simulation examples illustrating the application of the proposed Entropy paradigm to the multi-model epidemic system discussed in Section 4. Thus, the behavior of Algorithms 1 and 2 will be shown in this section through numerical examples in open and closed-loop. The accurate model considered as the one generating the actual or true data is the SEIR one described in [27] with vaccination:

(51) $$\begin{array}{c} \frac{dS}{dt}=\mu -\beta (t)S(t)I(t)-\mu S(t)+\delta R(t)-V(t)\\ \frac{dE}{dt}=\beta (t)S(t)I(t)-(\mu +\varepsilon )E(t)\\ \frac{dI}{dt}=\varepsilon E(t)-(\mu +\gamma )I(t)\\ \frac{dR}{dt}=\gamma I(t)-(\mu +\delta )R(t)+V(t) \end{array}$$

where μ=2.0 years^−1^ is the growth and death rate of the population, ε=1.0 years^−1^, δ=0.1 days^−1^, γ=0.02 days^−1^ are the instantaneous per capita rates of leaving the exposed, infected and recovered stages, respectively, and V(t) denotes the vaccination. This model fits in the structure given by (26) where u(t)=V(t) acts as the control command. The initial conditions are given by S(0)=E(0)=I(0)=R(0)=0.1. All the parameters are assumed to be constant except β(t), the disease transmission coefficient, which describes the seasonality in the infection rate and is given by the widely accepted Dietz’s model, [30], $\beta (t)=\beta_{0}\left(1+bcos(2\pi t)\right)$ with $\beta_{0}=6.2$ and b=0.6. The function β(t) describes annual seasonality in this example. Figure 1 shows the behavior of this system in the absence of any external action (i.e., in open loop). As it can be observed in Figure 1, the disease is persistent since the infectious do not converge to a zero steady-state value asymptotically. This situation will be tackled in Section 5.2 by means of the vaccination function in order to generate a closed-loop system whose infectious tend to zero. 

**Figure 2:**
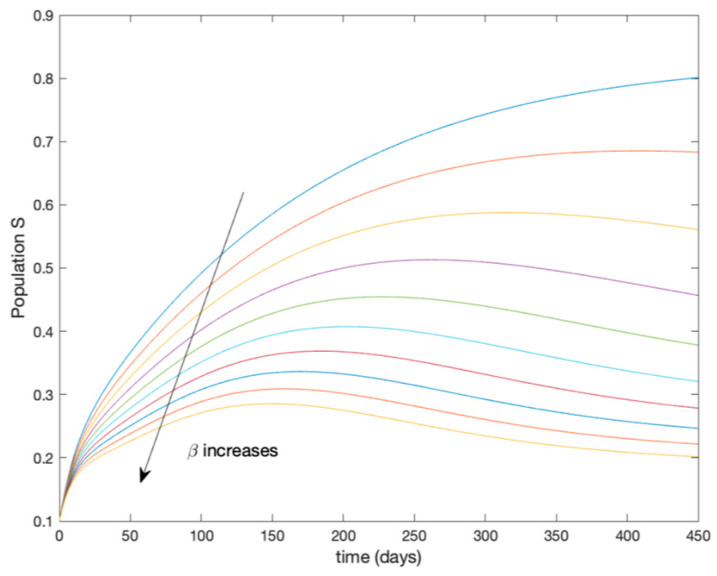
Dynamics of susceptible (S) for different values of constant β.

**Figure 3:**
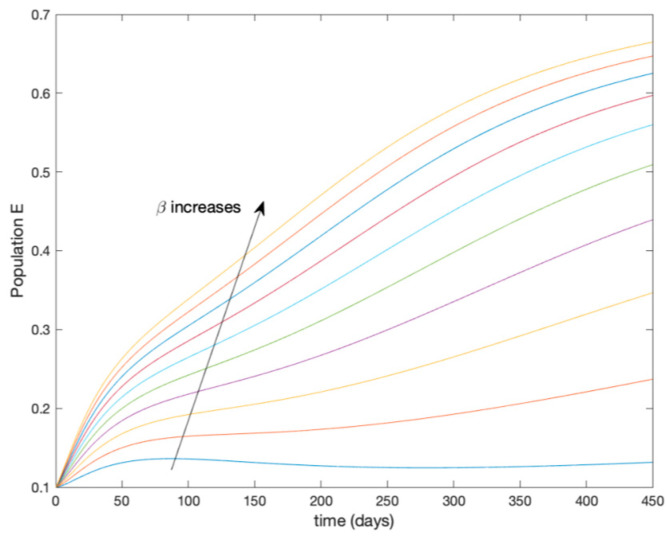
Dynamics of exposed (E) for different values of constant β.

**Figure 4:**
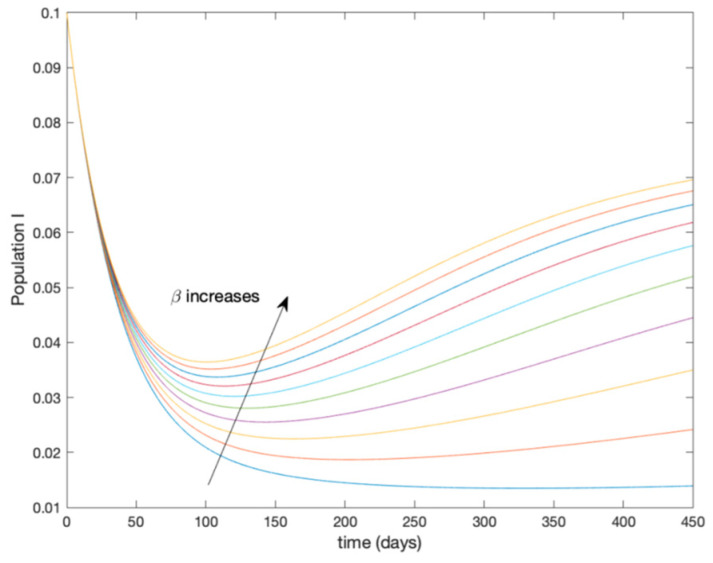
Dynamics of infectious (I) for different values of constant β.

**Figure 5:**
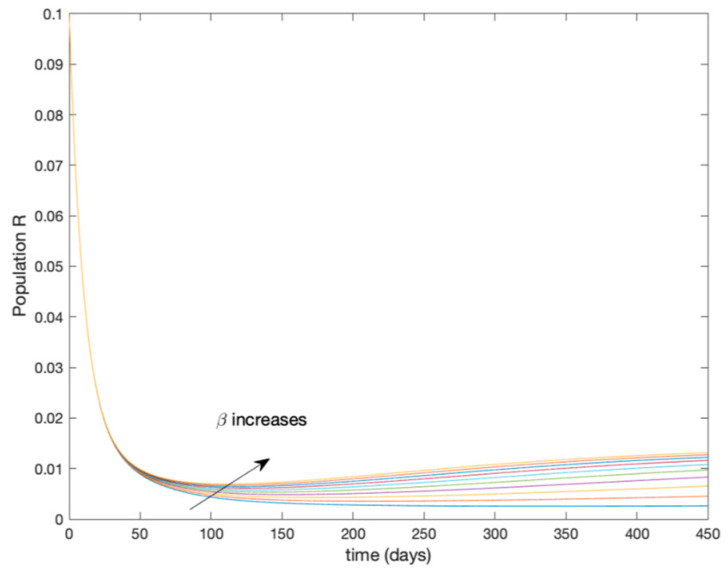
Dynamics of immune (R) for different values of constant β.

This accurate time-varying model is described by a number of simplified time-invariant models running in parallel with the same constant parameter values and fixed values of β obtained according to (44). In this example Q=9, so that 10 models will be running in parallel. The Figure 2, Figure 3, Figure 4 and Figure 5 show the trajectory followed by these fixed models for the different considered constant values of β.

It is worth to mention that none of the models whose trajectory is depicted in Figure 2, Figure 3, Figure 4 and Figure 5 can solely describe the dynamics of the whole accurate model, since none of them is able to reproduce the complex behavior generated by the time-varying seasonal incidence rate, β(t). In this way, the switching mechanisms given by Algorithms 1 and 2 inspired in the entropy paradigm are employed to generate a switched piecewise constant model able to describe the time-varying system by means of time-invariant ones. Section 5.1 shows the capabilities of the switched model to reproduce the behavior of the accurate model in open loop, i.e., in the absence of any external action.

### 5.1. Open-Loop Switched Model

**Figure 6:**
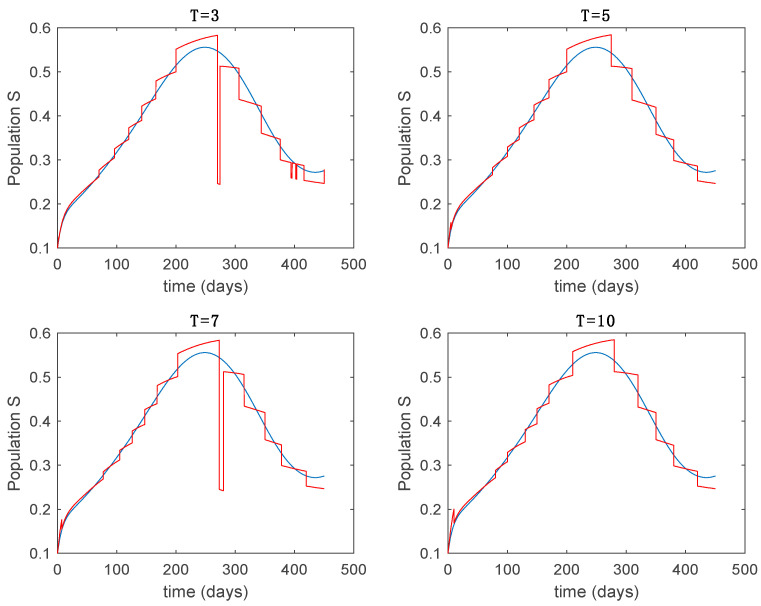
Trajectory of the susceptible for the switched system (Algorithm 1) and different values of T.

**Figure 7:**
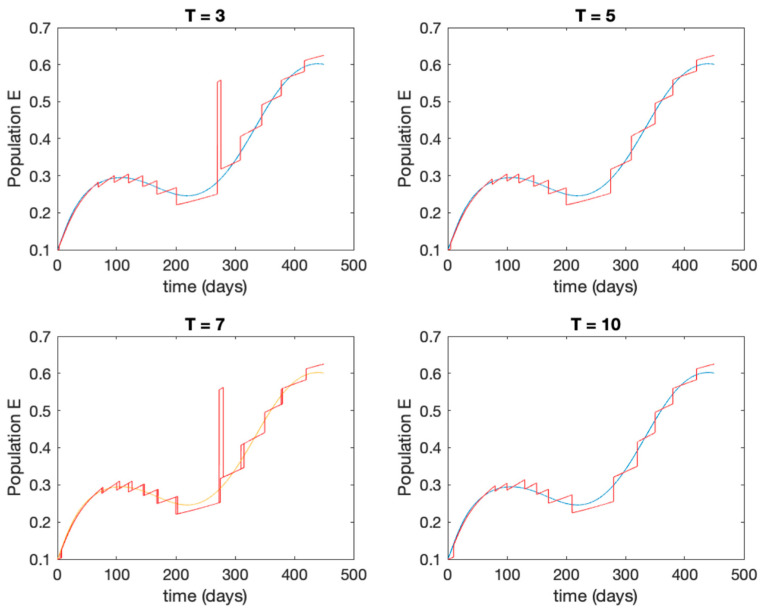
Trajectory of the exposed for the switched system (Algorithm 1) and different values of T.

**Figure 8:**
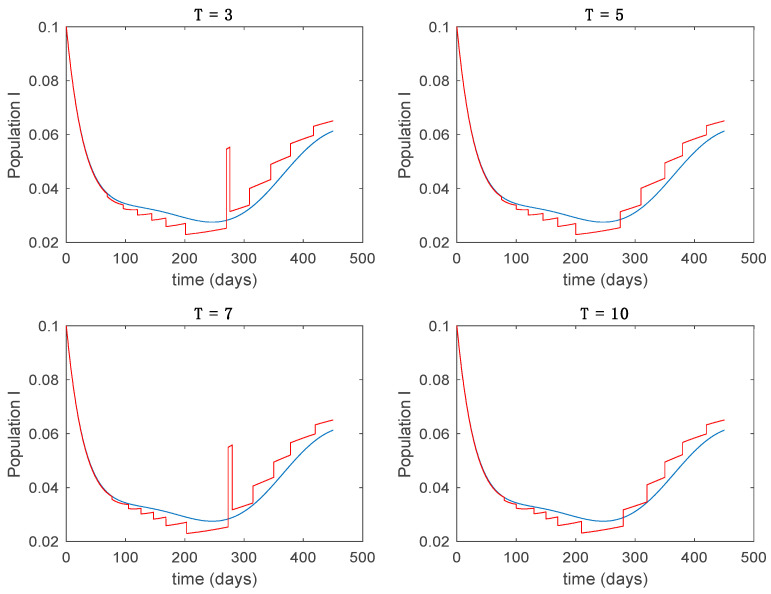
Trajectory of the infectious for the switched system (Algorithm 1) and different values of T.

**Figure 9:**
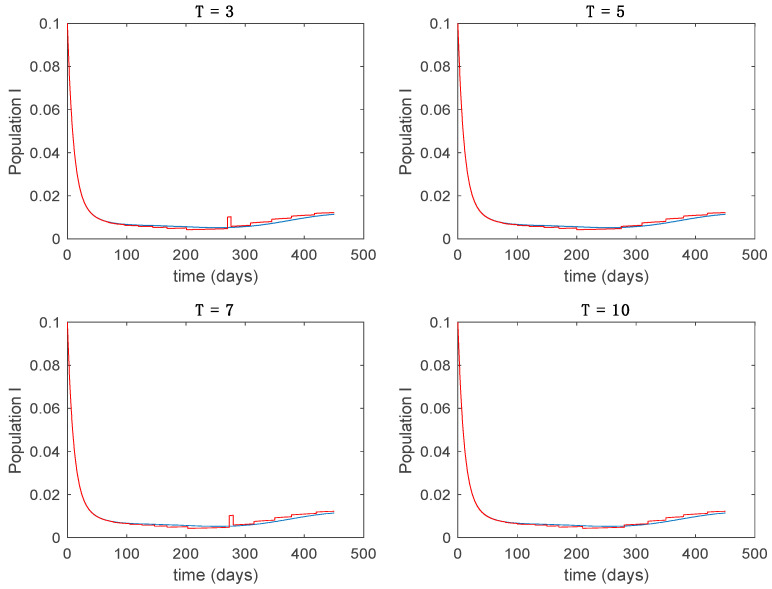
Trajectory of the immune for the switched system (Algorithm 1) and different values of *T*.

**Figure 10:**
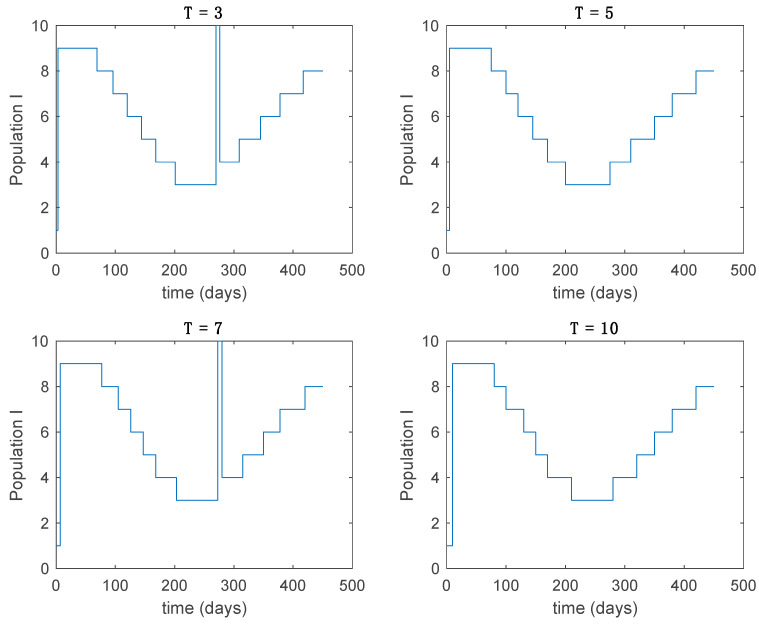
Active model within time intervals for the different values of *T* (Algorithm 1).

**Figure 11:**
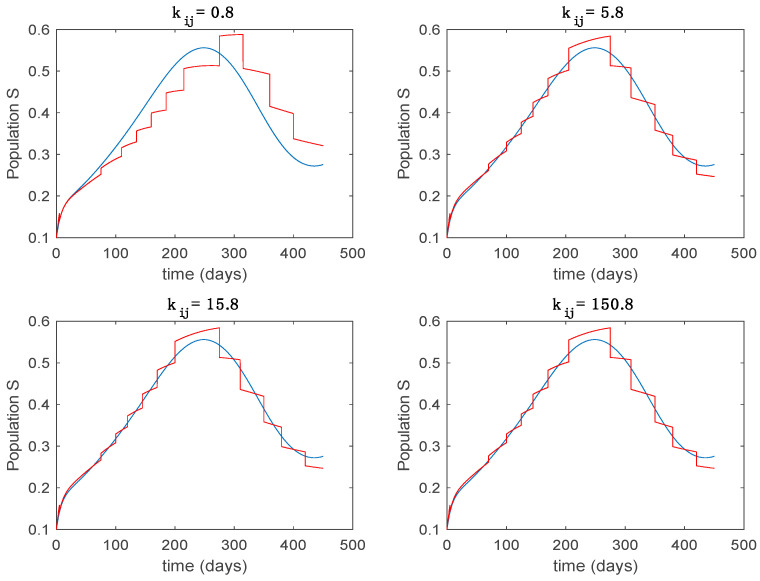
Trajectory of the susceptible for the switched system (Algorithm 1) and different values of $k_{ij}$.

**Figure 12:**
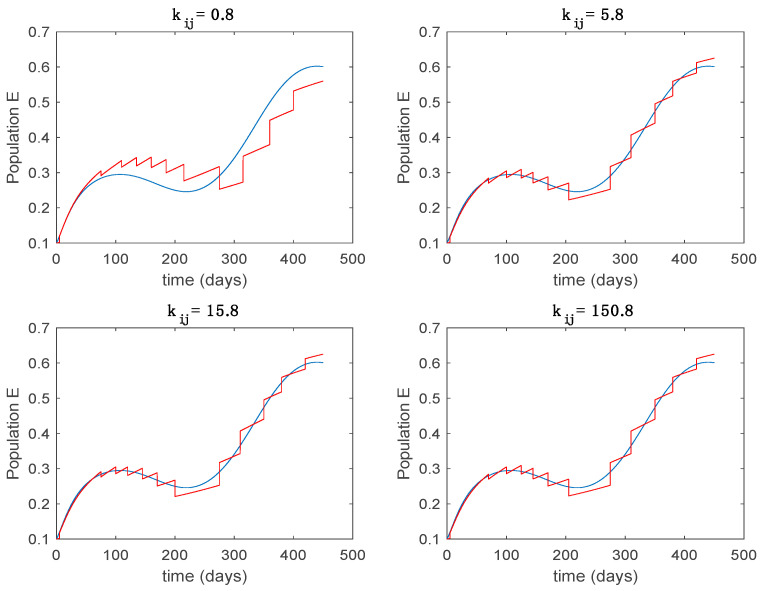
Trajectory of the exposed for the switched system (Algorithm 1) and different values of $k_{ij}$.

**Figure 13:**
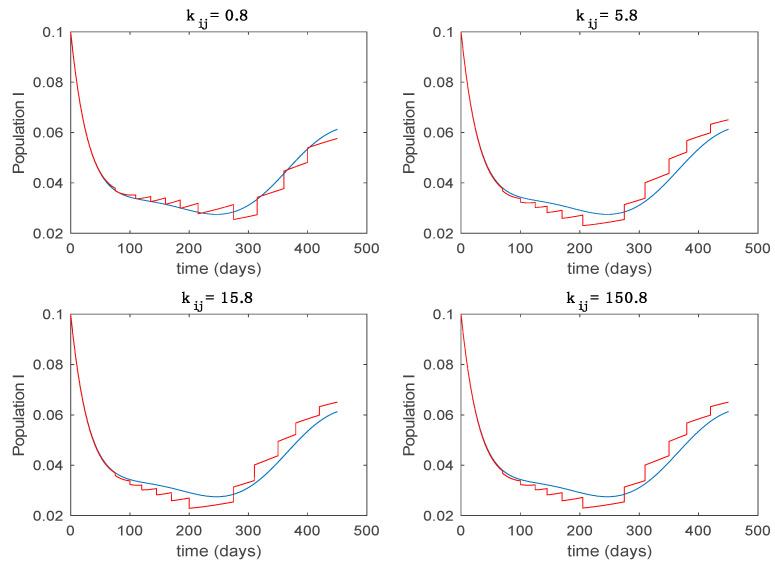
Trajectory of the infectious for the switched system (Algorithm 1) and different values of $k_{ij}$.

**Figure 14:**
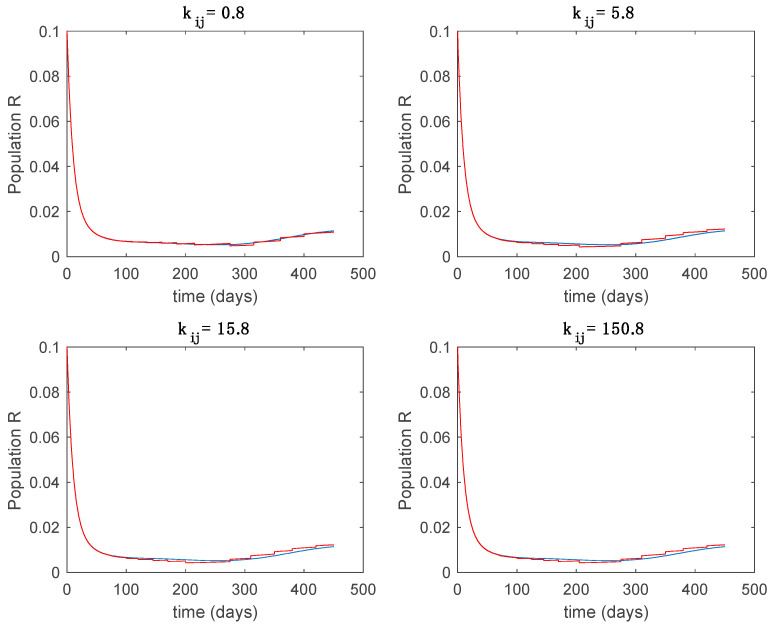
Trajectory of the immune for the switched system (Algorithm 1) and different values of $k_{ij}$.

**Figure 15:**
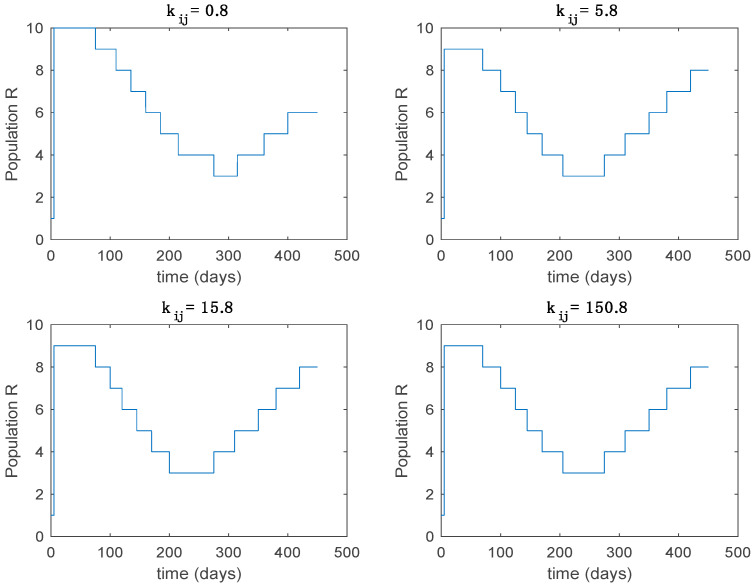
Active model within each time interval for different values of $k_{ij}$ (Algorithm 1).

**Figure 16:**
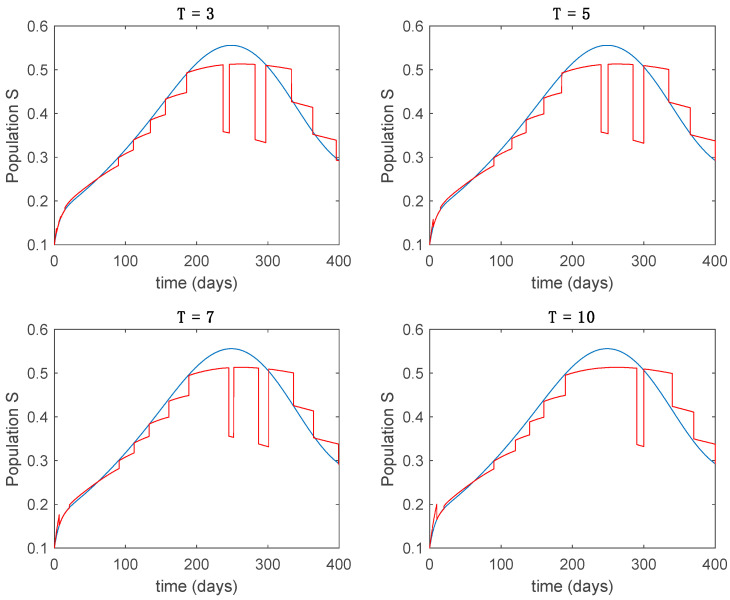
Trajectory of the susceptible for the switched system (Algorithm 2) and different values of T.

**Figure 17:**
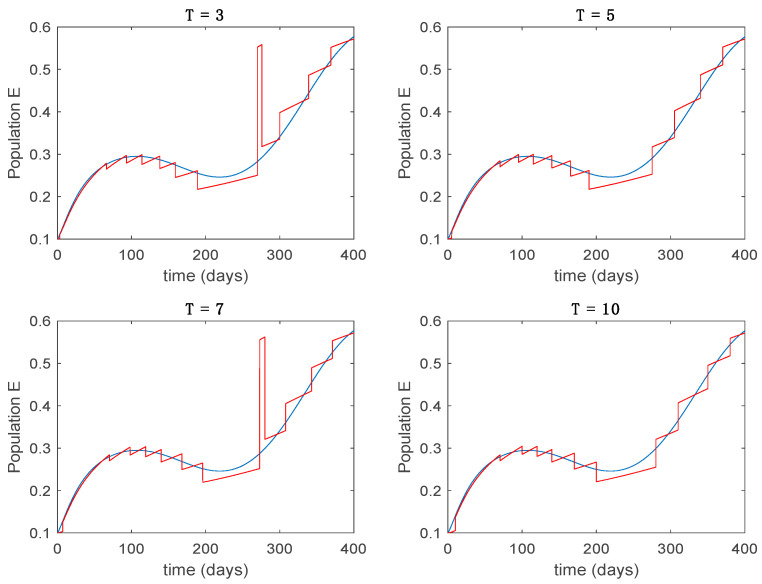
Trajectory of the exposed for the switched system (Algorithm 2) and different values of T.

**Figure 18:**
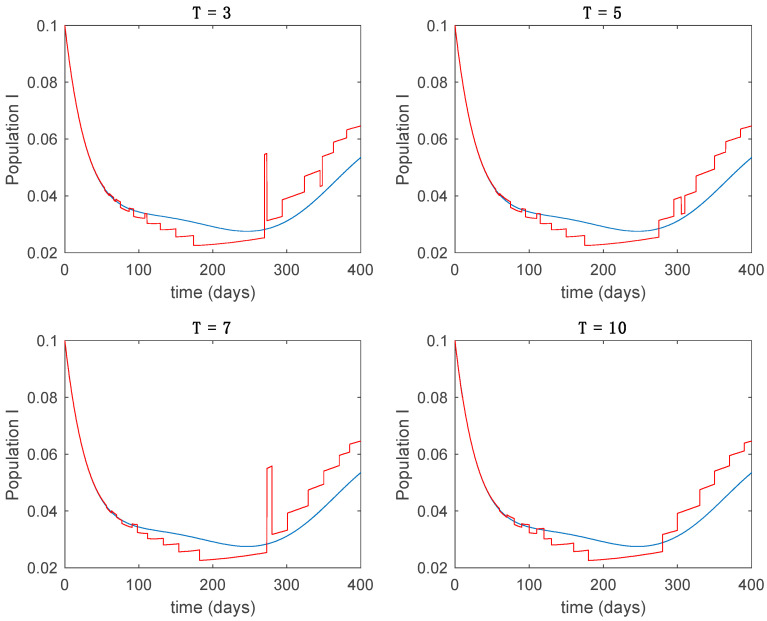
Trajectory of the infectious for the switched system (Algorithm 2) and different values of T.

**Figure 19:**
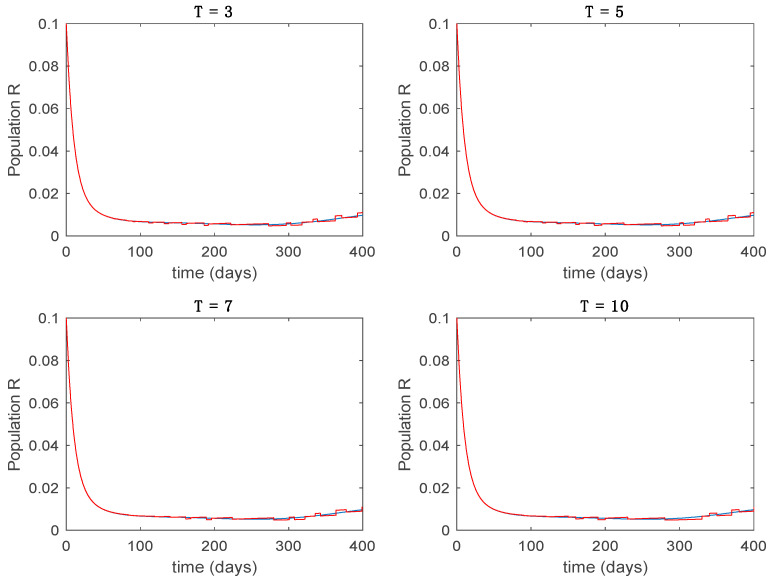
Trajectory of the immune for the switched system (Algorithm 2) and different values of T.

**Figure 20:**
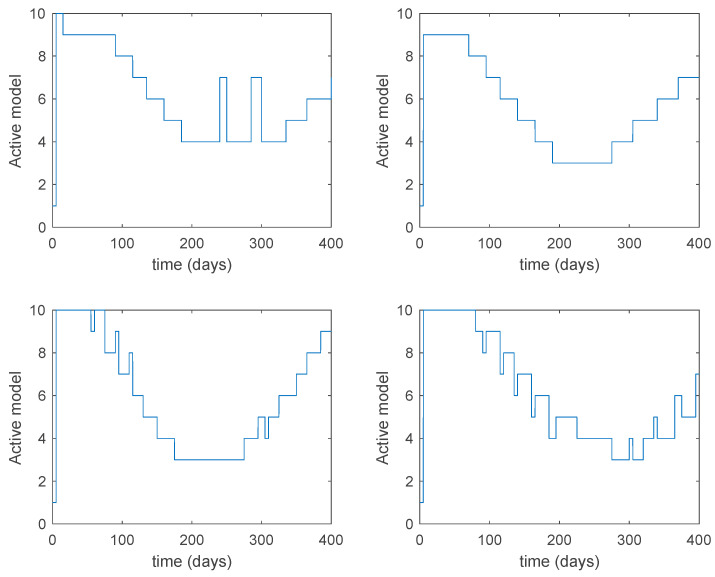
Active model within each time interval for Algorithm 2 and values T=5 days and $k_{ij}=15.8$.

**Figure 21:**
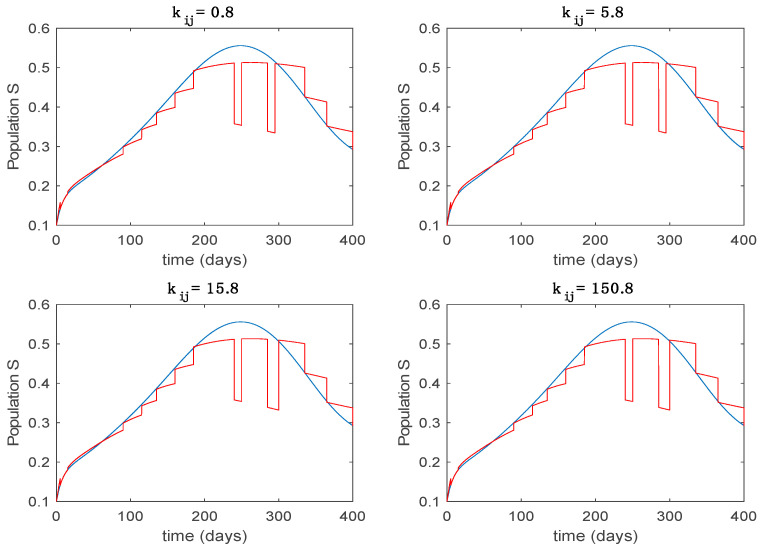
Trajectory of the susceptible for the switched system (Algorithm 2) and different values of $k_{ij}$.

**Figure 22:**
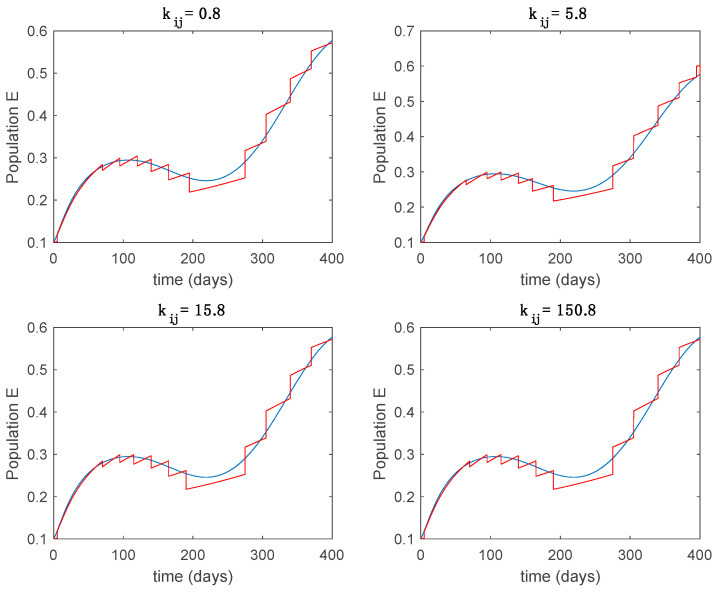
Trajectory of the exposed for the switched system (Algorithm 2) and different values of $k_{ij}$.

**Figure 23:**
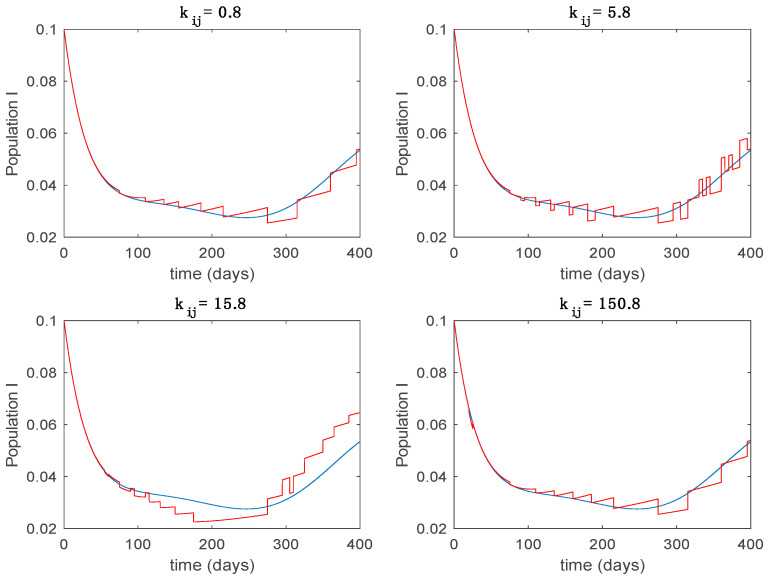
Trajectory of the infectious for the switched system (Algorithm 2) and different values of $k_{ij}$.

**Figure 24:**
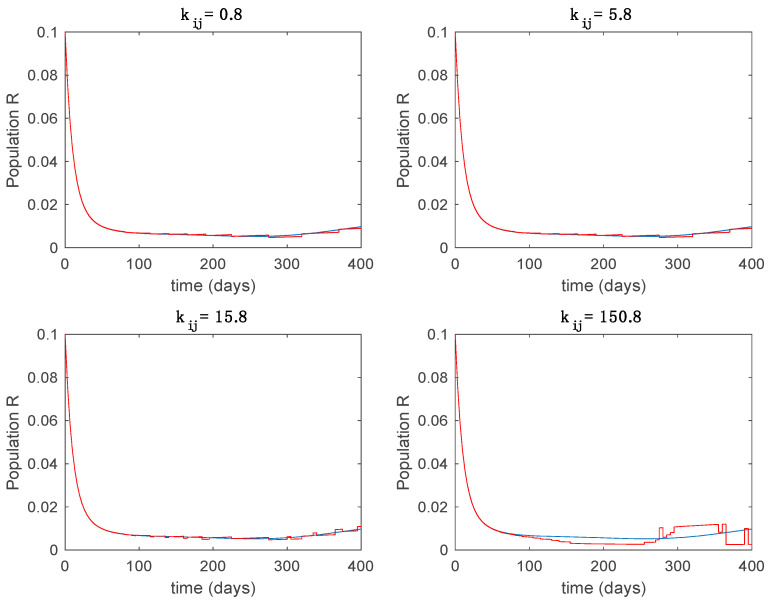
Trajectory of the immune for the switched system (Algorithm 2) and different values of $k_{ij}$.

In this subsection the vaccination function is set to zero so that all the models are running in open loop. The simulations are completed for different values of the switching time T (in days) and the probability gains $k_{ij}$ of (49) in order to observe their influence on the open-loop trajectories. The values of the probability gains include the case of inflation as well. Thus, the Figure 6, Figure 7, Figure 8, Figure 9 and Figure 10 display the trajectories of the switched system for Algorithm 1, different values of the switching time T and a constant gain of $k_{ij}=15.8$ while Figure 11, Figure 12, Figure 13, Figure 14 and Figure 15 depict the trajectories for different values of the probability gains $k_{ij}$ and a constant value of T=5 days. In all the subsequent figures, the blue line denotes the output of the time-varying system while the output of the switched system is displayed in red; legends have been then omitted from figures for the sake of clarity. On the other hand, Figure 16, Figure 17, Figure 18, Figure 19, Figure 20, Figure 21, Figure 22, Figure 23 and Figure 24 display the trajectories under the same conditions but for Algorithm 2. 

From the above figures we can conclude that the switched system, either with Algorithm 1 or with Algorithm 2, is able to reproduce the shape of the trajectories of the accurate-time-varying system. Consequently, the presented approach is useful to describe the overall dynamics of a complex set of data, coming either from real measurements or from a complex model, by means of a simpler piece-wise time-invariant model. The switching rules based on the entropy of the errors between each model and the data have revealed to be an adequate frame for such a task. Figure 10 and Figure 15 show the active model selected within each time interval by Algorithm 1 to parameterize the switched system. Moreover, Figure 20 shows the active model when Algorithm 2 is employed. It is seen in Figure 20 how the algorithm selects a different model for each one of the state components of the system, which is the particularity of the algorithm. From the simulation results we can also observe that the probability value has a slight influence on the approximation performance and the selection of an appropriate value is not that critical. However, the switching time plays a more important role in the obtained performance as it can be deduced from the above examples. Moreover, there are no specific criteria for the selection of the switching time while a trial-error work can be performed in order to obtain an appropriate value for it. Finally, it can also be observed that Algorithm 1 is able to attain closer curves to the actual system than Algorithm 2, especially when it comes to the Susceptible population. The above figures show that the disease is persistent in the sense that the infectious do not vanish with time. Therefore, in the next Section 5.2 vaccination is used as external control to make the infectious converge to zero asymptotically.

### 5.2. Closed-Loop Control

**Figure 25:**
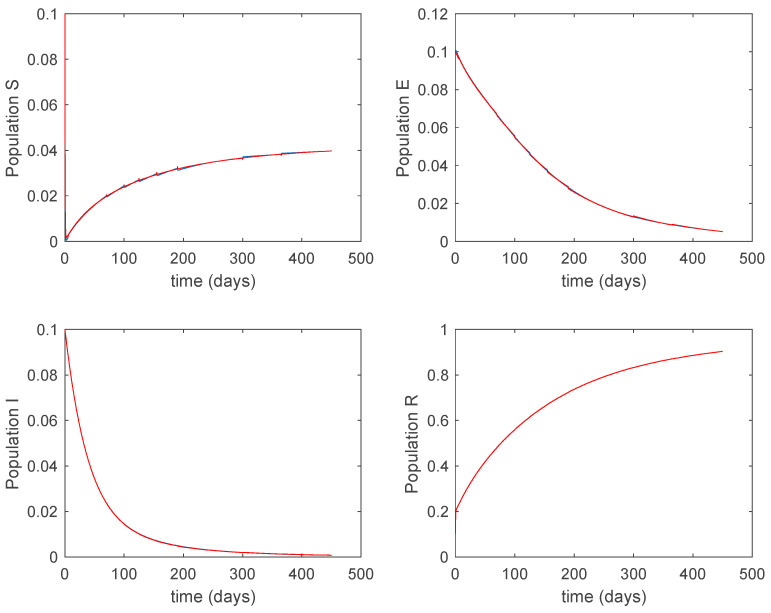
Evolution of the closed-loop system when vaccination is applied and Algorithm 1 is used.

**Figure 26:**
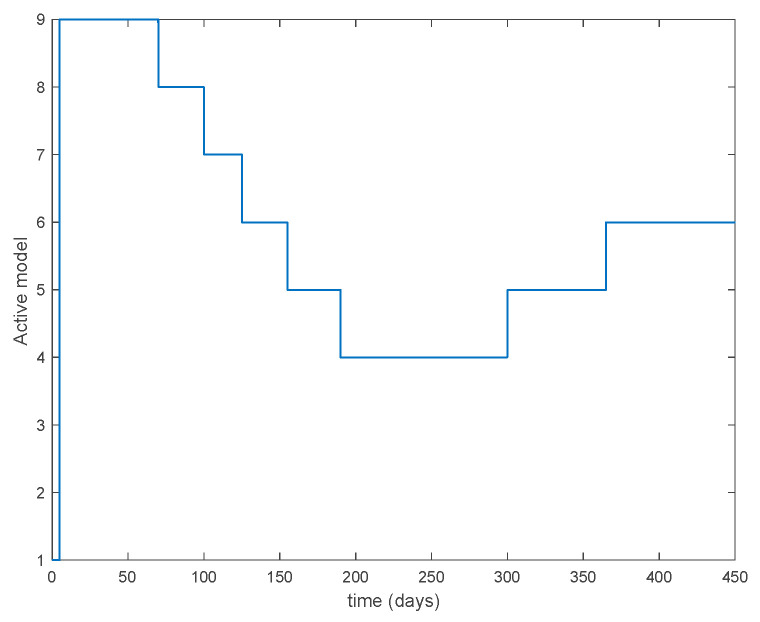
Active model when Algorithm 1 is employed.

**Figure 27:**
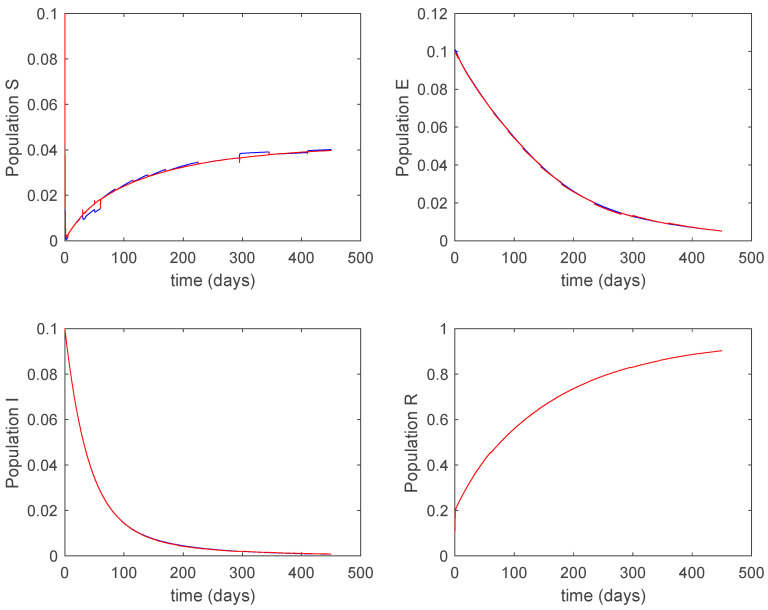
Evolution of the closed-loop system when vaccination is applied and Algorithm 2 is used.

**Figure 28:**
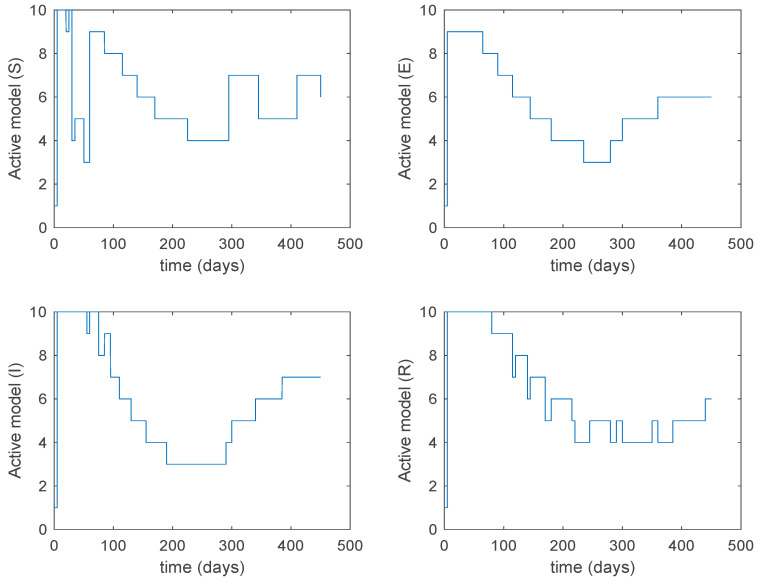
Active model when Algorithm 2 is employed.

**Figure 29:**
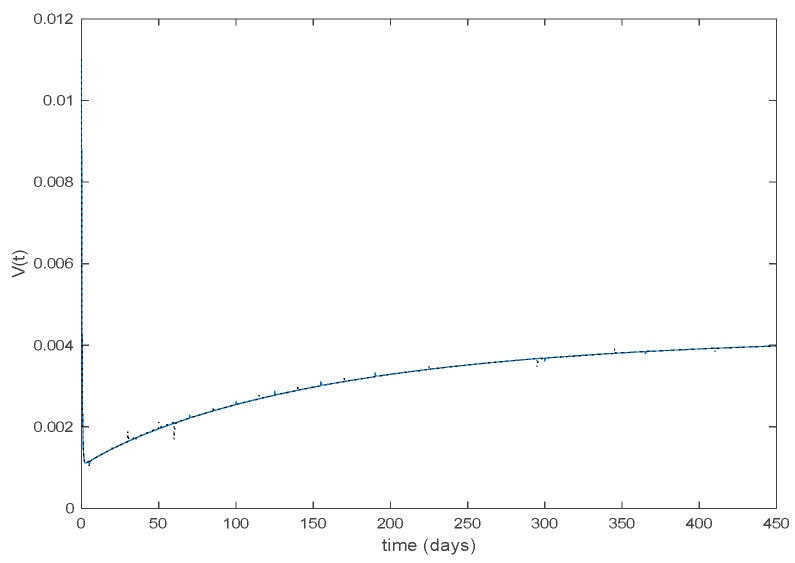
Vaccination function for Algorithms 1 and 2.

**Figure 30:**
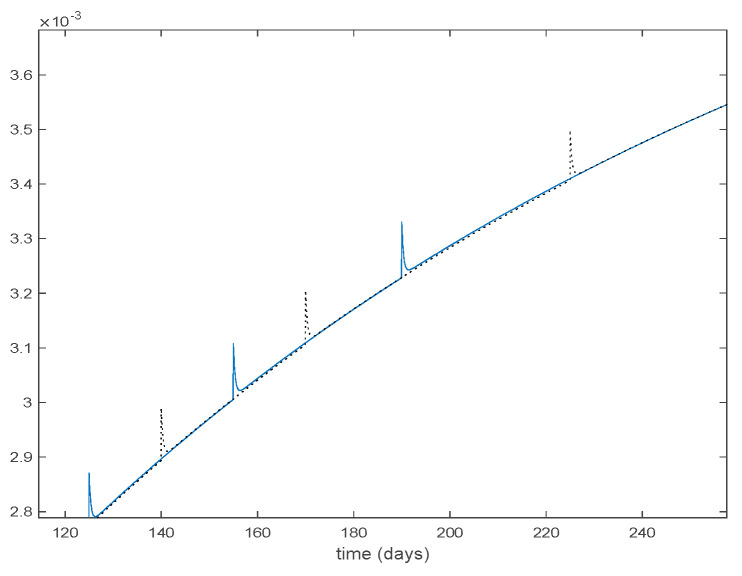
Zoom on the vaccination function for Algorithms 1 and 2.

In this subsection, vaccination is employed to avoid the persistency of the disease. To this end, the following state-feedback type vaccination law is used [29]:

$$V(t)=K_{S}S_{active}(t)+K_{I}I_{active}(t)$$

with $K_{S}=0.1$ and $K_{I}=0.01$ being the state-feedback gains and $S_{active}(t)$, $I_{active}(t)$ the state components of the corresponding active model according to Algorithms 1 and 2. Along this section, T=5 days and $k_{ij}=15.8$. Figure 25 displays the evolution of the closed-loop system when Algorithm 1 is employed while Figure 26 shows the corresponding active model selected to parameterize the control command within each time interval. Figure 27 shows the trajectories for Algorithm 2 and Figure 28 shows the corresponding active model selected to parameterize the control command within each time interval. From Figure 25 and Figure 27 we can conclude that the output of the real system and the output of the active model are practically the same when a feedback control action is included in the system. Thus, the approximation errors appearing in open loop vanish in closed-loop because of the control action. Moreover, there are no differences in the closed-loop trajectories generated by Algorithms 1 and 2. It can also be observed in Figure 25 and Figure 27 that the infectious tend to zero eradicating the disease from the population, as desired. Therefore, the control objective is achieved. Figure 29 and Figure 30 display the vaccination function calculated by using both algorithms. It can be seen that both control commands are very similar with only some peaks associated with the switching process making the difference between one and another. Overall, the proposed approach has been showed to be a powerful tool to model the complex time-varying system.

### 5.3. Example with Actual Data

In this subsection the proposed entropy approach is applied to the real case of measles in the city of New York. In this way, it was stated in [33] that measles outbreaks in NYC during the period 1930–1970 can be appropriately described by a SIR model with a time-varying contact rate. Moreover, [33] estimates a monthly contact rate for the model while [34] proposes a Dietz-type contact rate for this problem. Thus, this real situation fits in with the formulation treated in the application problem. The authors of [34] gathered the weekly amount of reported measles cases for 93 years corresponding to the period 1891–1984 and made them publicly available as a supplementary material of [34]. In this simulation, the Algorithm 2 from Section 4 will be used to generate a switched model describing the data set of the year 1960. The SIR model describing the problem is given by:

(52) $$\begin{array}{c} \frac{dS}{dt}=\nu -\beta (t)SI-\mu S\\ \frac{dI}{dt}=\beta (t)SI-(\gamma +\mu )I\\ \frac{dR}{dt}=\gamma I-\mu R \end{array}$$

**Figure 31:**
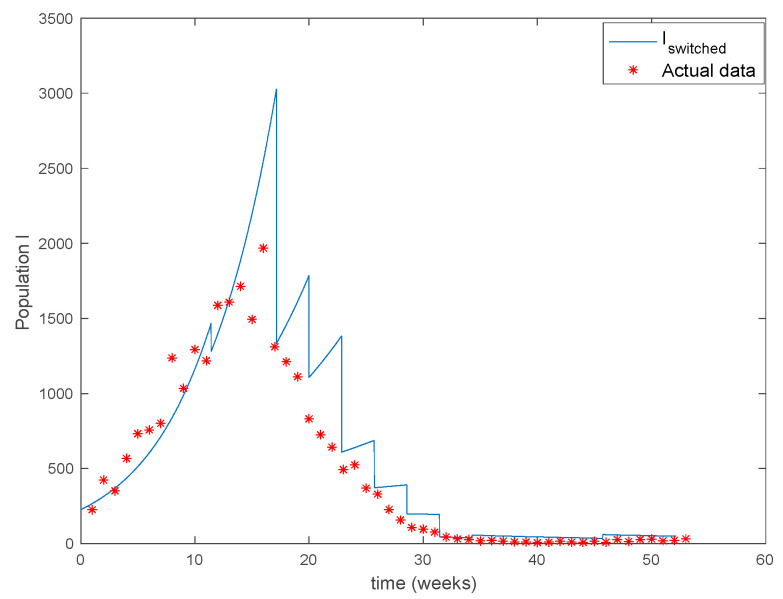
Comparison of the output of the switched model with the actual data set for 1960.

The parameters of the model are estimated in [34] to be $\gamma^{-1}=13$ days, $\mu =0.02yr^{-1}$, $\beta_{\min}=1.79\cdot 10^{-10}$, $\beta_{\max}=5.3831\cdot 10^{-10}$ and the yearly number of new born is approximately $10^{5}$. The initial values of the populations are S(0)=7782000, the population of New York in 1960, I(0)=225 and R(0)=0 while T=20 days. There are 40 time-invariant models linearly spaced between $\beta_{\min}$ and $\beta_{\max}$. Actual data for the infectious are used in (49) in order to calculate the error corresponding to each one of models running in parallel. The Figure 31 shows the number infectious predicted by the switched model compared to the actual data during 1960. The *x* axis of the figure represents the 52 weeks of the year.

**Figure 32:**
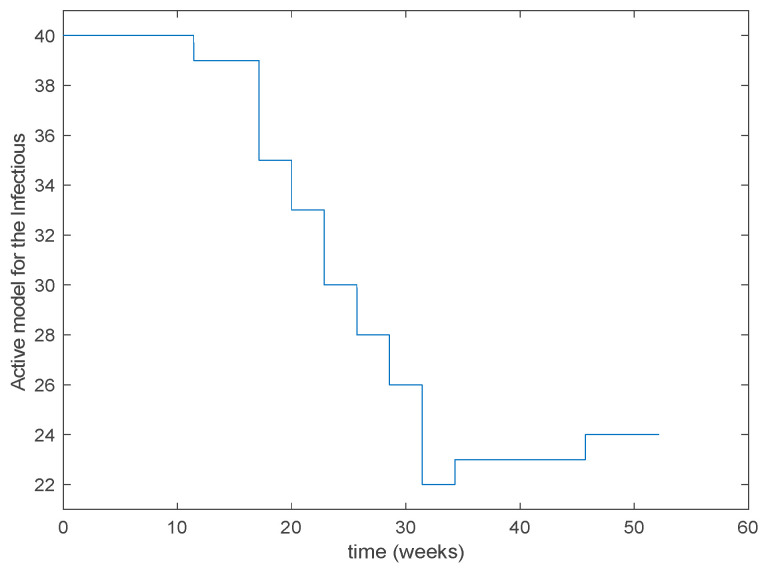
Active model selected at each time interval for the infectious population.

As it can be observed from Figure 31 the proposed approach succeeds at reproducing the trend contained in the data and predicting the time when the outbreak reaches the peak. Finally, the Figure 32 shows the active model at each time for the infectious population. Since the data are only available for the infectious, only this population has been displayed in the figures.

## 6. Conclusions

This paper has presented results for the Shannon entropy when a complete the events of a complete finite and discrete set are eventually subject to relative errors of their associate probabilities. As a result, the current system of events, eventually under probabilistic errors, can lose its completeness since it may be either deflated or inflated in the sense that the total probability for the whole sets might be either below or beyond unity. Later on the previous technical results have been applied to control of epidemics evolution in the case that either the disease transmission coefficient rate is not well-known or it varies through time due, for instance, to seasonality. For such a purpose, a finite predefined set of running models, described by coupled differential equations, is chosen which covers a range of variation of such a coefficient transmission rate within known lower-bound and upper-bound limits. Each one of such models is driven by a constant disease transmission rate and the whole set of models covers the whole range of foreseen variation of such a parameter in the real system. In a general context, different uncertain parameters, or groups of parameters, other that the coefficient transmission rate could be checked by the proposed minimum-error entropy supervisory scheme. Two monitoring algorithms have been proposed to select the active one which minimizes the accumulated entropy of the absolute error data/model within each supervision time interval. A switching rule allows to choose another active model as soon as it is detected that the current active model becomes more uncertain than other (s) related to the observed data. Some numerical results have been also performed and discussed including a discussion on a real registered case. The active model, which is currently in operation, generates the vaccination and/or treatment controls to be injected to the real epidemic process.
